# Global analysis of WRKY transcription factor superfamily in *Setaria* identifies potential candidates involved in abiotic stress signaling

**DOI:** 10.3389/fpls.2015.00910

**Published:** 2015-10-26

**Authors:** Mehanathan Muthamilarasan, Venkata S. Bonthala, Rohit Khandelwal, Jananee Jaishankar, Shweta Shweta, Kashif Nawaz, Manoj Prasad

**Affiliations:** National Institute of Plant Genome ResearchNew Delhi, India

**Keywords:** WRKY transcription factors, *Setaria italica*, *Setaria viridis*, abiotic stress, stress signaling, expression profiling, comparative mapping

## Abstract

Transcription factors (TFs) are major players in stress signaling and constitute an integral part of signaling networks. Among the major TFs, WRKY proteins play pivotal roles in regulation of transcriptional reprogramming associated with stress responses. In view of this, genome- and transcriptome-wide identification of WRKY TF family was performed in the C_4_model plants, *Setaria italica* (SiWRKY) and *S. viridis* (SvWRKY), respectively. The study identified 105 SiWRKY and 44 SvWRKY proteins that were computationally analyzed for their physicochemical properties. Sequence alignment and phylogenetic analysis classified these proteins into three major groups, namely I, II, and III with majority of WRKY proteins belonging to group II (53 SiWRKY and 23 SvWRKY), followed by group III (39 SiWRKY and 11 SvWRKY) and group I (10 SiWRKY and 6 SvWRKY). Group II proteins were further classified into 5 subgroups (IIa to IIe) based on their phylogeny. Domain analysis showed the presence of WRKY motif and zinc finger-like structures in these proteins along with additional domains in a few proteins. All *SiWRKY* genes were physically mapped on the *S. italica* genome and their duplication analysis revealed that 10 and 8 gene pairs underwent tandem and segmental duplications, respectively. Comparative mapping of *SiWRKY* and *SvWRKY* genes in related C_4_ panicoid genomes demonstrated the orthologous relationships between these genomes. *In silico* expression analysis of *SiWRKY* and *SvWRKY* genes showed their differential expression patterns in different tissues and stress conditions. Expression profiling of candidate *SiWRKY* genes in response to stress (dehydration and salinity) and hormone treatments (abscisic acid, salicylic acid, and methyl jasmonate) suggested the putative involvement of *SiWRKY066* and *SiWRKY082* in stress and hormone signaling. These genes could be potential candidates for further characterization to delineate their functional roles in abiotic stress signaling.

## Introduction

Plants are exposed to diverse environmental stresses, which significantly affect their growth and development leading to drastic decrease in productivity. Among the different environmental stimuli, abiotic stresses are predominant, which includes drought, heat, salinity, and submergence. Climate change due to global warming is another aggravating challenge that influences the sustainability and productivity of crop plants (Kole et al., 2015). Plants have developed broad-spectrum defense responses to circumvent these stresses and exhibit stress tolerance or stress avoidance through acclimation and adaptation mechanisms (Mickelbart et al., 2015). On perception of stress, a complex signal transduction pathway (either abscisic acid-dependent or -independent) is induced, which initiates molecular, physiological and metabolic responses that ultimately enhance stress tolerance (Lata et al., 2015). Transcription factors (TFs) are a class of genes that predominate as tolerance determinants in plants (Mickelbart et al., 2015) by regulating the expression of stress-inducible genes. The TFs may constitute gene networks or signaling cascades, by which they regulate other TFs and/or other regulatory and/or functional genes (Tran and Mochida, 2010). Approximately 7% of the plant genome encodes for TFs (Udvardi et al., 2007), which are classified into 58 TF families (Jin et al., 2014). Among these TFs, WRKY is the seventh largest TF family (http://planttfdb.cbi.pku.edu.cn/). WRKY TFs are characterized by their unique WRKYGQK motif followed by a metal chelating zinc finger motif (CX_4−5_CX_22−23_HXH or CX_5−8_CX_25−28_HX_1−2_C) (Eulgem et al., 2000). These WRKY proteins bind to a specific domain called W-box in the promoter region with consensus sequence (C/T)TGAC[T/C], resulting in the expression of downstream target genes (Eulgem et al., 2000). In addition to W-box, WRKY TFs can also interact with a sugar responsive cis-element called SURE and activate transcription of downstream genes (Sun et al., 2003).

Several reports have shown the regulatory role of WRKY TFs in signaling pathways and modulation of diverse molecular and physiological processes including pollen development and function (Guan et al., 2014), seed dormancy (Rushton et al., 2010; Ding et al., 2014), seed development (Johnson et al., 2002; Sun et al., 2003; Luo et al., 2005), flowering time and plant height (Cai et al., 2014b), somatic embryogenesis (Alexandrova and Conger, 2002), biomass (Wang et al., 2010; Yu et al., 2013), secondary metabolite biosynthesis (Sun et al., 2003; Xu et al., 2004; Ma et al., 2009; Suttipanta et al., 2011), hormone signaling (Zhang et al., 2004) and leaf senescence (Miao et al., 2004). More importantly, WRKY TFs have been shown to get activated in response to different biotic (Dong et al., 2003; Muthamilarasan and Prasad, 2013) and abiotic stresses (Tang et al., 2013), including heat and drought (Rizhsky et al., 2002; Wu et al., 2009; Ren et al., 2010), cold (Huang and Duman, 2002; Pnueli et al., 2002), salinity (Jiang and Deyholos, 2006), wounding (Hara et al., 2000; Yoo et al., 2014), bacterial infection (Dellagi et al., 2000; Du and Chen, 2000; Chen et al., 2002; Chen and Chen, 2002; Deslandes et al., 2002; Kim et al., 2008), fungal invasion (Chen et al., 2002; Zheng et al., 2006; Marchive et al., 2007), virus attack (Wang et al., 1998; Yang et al., 1999; Chen et al., 2002, 2013; Huh et al., 2012) and defense against oomycetes (Beyer et al., 2001; Kalde et al., 2003).

Thus, considering the vital role of WRKY TFs in various molecular, biological and physiological processes, the *WRKY* gene family has been extensively characterized in various crop plants (Zhang and Wang, 2005), such as rice (Ross et al., 2007), cucumber (Ling et al., 2011), maize (Wei et al., 2012), tomato (Huang et al., 2012), Castor bean (Li et al., 2012), physic nut (Xiong et al., 2013), barley (Liu et al., 2014), *Brachypodium* (Wen et al., 2014), *Gossypium raimondii, G. hirsutum* (Cai et al., 2014a; Dou et al., 2014), grapevine (Wang et al., 2014), *G. arboretum* (Ding et al., 2015), cabbage (Yao et al., 2015), and in trees including rubber (Li et al., 2014), poplar (He et al., 2012; Jiang et al., 2014) and willow (Rao et al., 2015), and in *Arabidopsis* (de Pater et al., 1996; Deslandes et al., 2002; Song and Gao, 2014). However, no such studies have been reported in C_4_ models, *Setaria italica* (foxtail millet) and *S. viridis* (green foxtail). Both *S. italica* and its wild progenitor *S. viridis* have collectively been accentuated as model crops for expediting functional genomics studies in Panicoideae, particularly C_4_ photosynthesis, biofuel traits and abiotic stress tolerance (Brutnell et al., 2010, 2015; Li and Brutnell, 2011; Wang et al., 2011; Lata et al., 2013; Diao et al., 2014; Muthamilarasan and Prasad, 2015).

In view of their importance, the U.S. Department of Energy Joint Genome Institute and Beijing Genomics Institute, China have independently sequenced the genomes of *S. italica* and *S. viridis* (Bennetzen et al., 2012; Zhang et al., 2012). The availability of genome sequence information of *S. italica* in public domain has facilitated the identification of 2297 putative TFs belonging to 55 families (Bonthala et al., 2014). Of these 55 families, NAC (Puranik et al., 2012), AP2/ERF (Lata et al., 2014), MYB (Muthamilarasan et al., 2014a) and C_2_H_2_ zinc fingers (Muthamilarasan et al., 2014b) have been extensively characterized and their expression patterns in response to different abiotic stresses and hormone treatments have been investigated. However, no such global analysis of TFs has been performed in *S. viridis* due to non-availability of genome sequence in public domain (Muthamilarasan and Prasad, 2015). Recently, Xu et al. (2013) pooled the RNA isolated from *S. viridis* at three developmental stages, namely seed germination, vegetative growth, and reproduction in different tissues including leaf, stem, node, crown, root, spikelet, floret, and seed tissues. Subsequently, cDNA library was constructed from the pooled RNA and sequenced using Illumina HiSeq 2000 platform (Xu et al., 2013). Transcriptome-wide analysis of TFs has been demonstrated in important crop plants, namely barley (Tombuloglu et al., 2013), bread wheat (Okay et al., 2014), *Medicago sativa* (Postnikova et al., 2014), and *G. aridum* (Fan et al., 2015). In the present study, similar computational approach has been used to identify WRKY encoding transcripts from *S. viridis* transcriptome and the identified transcripts were analyzed with WRKY encoding genes of *S. italica*. Being the first comprehensive study on WRKY TFs in *S. italica* and *S. viridis*, the present study provides insights into the functional aspects of these TFs in response to abiotic stress, and highlights potential candidates for further characterization toward delineating their functional role in abiotic stress signaling.

## Materials and methods

### *In silico* mining of WRKY proteins from *Setaria italica and S. viridis*

The WRKY domain-containing protein sequences of *Setaria italica* and *S. viridis* were identified using the method of Plant Transcription Factor Database (Jin et al., 2014). *S. italica* protein sequences (v2.1) were retrieved from Phytozome v10.2 (Goodstein et al., 2012) and HMMER search was executed using the PFAM domain (PF03106) (Finn et al., 2011). The HMM profile generated with WRKY TFs of maize (Wei et al., 2012) and rice (Ross et al., 2007) were used to generate HMM profile and searched against the protein sequences of *S. italica* using HMMER (Finn et al., 2011). Both *de novo* and reference-based transcriptome sequences of *S. viridis* (kindly provided by Prof. Xin-Guang Zhu; Xu et al., 2013) were used to generate unique clusters using CD-Hit (Fu et al., 2012) with default parameters and the resultant sequences were subjected to ORF prediction using OrfPredictor (Min et al., 2005). The obtained peptide sequences were used for identification of WRKY domain-containing proteins using the methodology described for *S. italica*. The identified WRKY sequences were confirmed for the presence of PFAM domain PF03106 (WRKY DNA-binding domain) using HMMSCAN (http://www.ebi.ac.uk/Tools/hmmer/search/hmmscan) and ScanProsite (http://prosite.expasy.org/scanprosite/; de Castro et al., 2006). The identified SiWRKY protein sequences were searched using BLASTP against *S. italica* database (v2.1) of Phytozome v10.2 to retrieve corresponding genomic, transcripts and coding sequences along with their chromosomal positions.

### Protein features, multiple sequence alignment, and phylogenetic analysis

Protein features including molecular weight, isoelectric point (pI) and instability index were predicted using ProtParam tool of ExPASy (Gasteiger et al., 2005). Amino acid sequences of WRKY TFs belonging to *S. italica* (SiWRKY) and *S. viridis* (SvWRKY) were imported into BioEdit v7.2.5 (Hall, 1999) and multiple sequence alignment was performed using ClustalW at default parameters. The SiWRKY and SvWRKY sequences along with maize sequences (ZmWRKY; Wei et al., 2012) were imported into MEGA v6.06 (Tamura et al., 2013) to construct a phylogenetic tree by Neighbor-Joining method and the bootstrap test was performed with 1000 iterations.

### Prediction of gene structure and chromosomal locations

The coding sequences and genomic sequences of SiWRKY proteins were analyzed using GSDS web server v2.0 (Hu et al., 2015) to identify the positions of introns and exons. Gene structure analysis for *SvWRKY* genes was not performed due to non-availability of genomic sequence of *S. viridis* in public databases. The information about chromosomal position of each *SiWRKY* gene was imported into MapChart v2.2 (Voorrips, 2002) and a physical map was constructed by mapping the genes in ascending order from short-arm telomere to long-arm telomere. MCScanX was used to identify tandem and segmental duplications of *SiWRKY* genes (Wang et al., 2012).

### Gene ontology annotation and promoter analysis

SiWRKY and SvWRKY amino acid sequences were analyzed using Blast2GO v3.0.10 (Conesa et al., 2005) to obtain gene ontology (GO) annotation. The sequences were screened using BLASTN against *Oryza sativa* protein sequences following which, mapping, InterProScan, and annotation were performed. GO enrichment was conducted using BiNGO plugin of Cytoscape v2.6 based on Benjamini and Hochberg false discovery correction value (*Q*-value) at 0.05 for the genes (Shannon et al., 2003; Maere et al., 2005). The *SiWRKY* gene sequences were searched using BLASTN against *S. italica* database in Phytozome to retrieve 2 kb upstream sequences. These sequences were screened for cis-regulatory elements using PLACE web server (Higo et al., 1999).

### Identification of orthologs in C_4_ grass genomes and Ks dating

Orthologous genes of *SiWRKY* and *SvWRKY* in sequenced C_4_ grasses including switchgrass (*Panicum virgatum*), sorghum (*Sorghum bicolor*), and maize (*Zea mays*) were identified by BLAST analysis of the gene and protein sequences, respectively against these genomes. Sequences with >90% similarity were used for performing reciprocal BLAST and potential orthologs were identified. A comparative map was constructed using Circos (Krzywinski et al., 2009). Synonymous (Ks) and non-synonymous (Ka) substitution rates were calculated for paralogous and orthologous genes by PAL2NAL server (http://www.bork.embl.de/pal2nal/) and period of divergence was calculated using the equation *T* = Ks/2λ, where λ was taken as 6.5 × 10^−9^ (Mishra et al., 2013; Puranik et al., 2013).

### *In silico* expression profiling of *SiWRKY* and *SvWRKY* genes

The transcriptome data of root (SRX128223), stem (SRX128225), leaf (SRX128224), spica (SRX128226), dehydration stress library (SRR629694), and control library (SRR629695) of *S. italica* were retrieved from European Nucleotide Archive (http://www.ebi.ac.uk/ena) (Zhang et al., 2012; Qi et al., 2013). *S. viridis* transcriptome data of pooled RNA isolated from samples across three developmental stages, namely seed germination, vegetative growth, and reproduction in different tissues including leaf, stem, node, crown, root, spikelet, floret, and seed tissues available under the accession number SRP019744 (Xu et al., 2013) was retrieved from DNA Data Bank of Japan (Tateno et al., 2002). The reads were filtered using NGS Toolkit (Patel and Jain, 2012), mapped on *S. italica* genome using CLC Genomics Workbench v4.7.1 and normalized by RPKM method. A heatmap was generated using MultiExperiment Viewer (MeV) v4.9 (Saeed et al., 2003).

### Expression profiling of candidate genes under abiotic stress and hormone treatments

Candidate *SiWRKY* genes were chosen for qRT-PCR expression analysis based on their *in silico* expression patterns. Primers were designed for the 3′ UTR of each transcript using GenScript Real-time PCR Primer Design tool (https://www.genscript.com/ssl-bin/app/primer) (Supplementary Table S1). *S. italica* cv. “Prasad” was chosen for the study as the cultivar was reported to be tolerant to salinity and dehydration stress (Lata et al., 2010; Puranik et al., 2011). The seeds were grown in green house following conditions described by Lata et al. (2014). Twenty-one day old seedlings were treated with 250 mM NaCl (salinity) and 20% PEG 6000 (dehydration) for abiotic stress, and 100 μM methyl jasmonate (MJ), 100 μM salicylic acid (SA), and 100 μM abscisic acid (ABA) for hormone treatments (Lata et al., 2014). Samples were collected at 0 h (control), 1 h (early), and 24 h (late) intervals, immediately frozen in liquid nitrogen and stored at −80°C. Total RNA from each sample was isolated following the method described by Logemann et al. (1987) and treated with RNase-free DNase I (50 U/ml). The quality and purity of RNA was tested using NanoDrop Spectrophotometer (Thermo Fisher Scientific, USA) [OD_260_:OD_280_ nm absorption ratio (1.8–2.0)] and integrity was checked by resolving on 1.2% agarose gel containing 18% formaldehyde. First strand complementary DNA was synthesized with random primers from 1 μg total RNA using Thermo Scientific Verso cDNA Synthesis kit (Thermo Fisher Scientific, USA) following manufacturer's instructions. qRT-PCR was performed in StepOne Real-Time PCR Systems (Applied Biosystems, USA). A constitutive *Act2* gene-based primer was used as the endogenous control (Kumar et al., 2013). The PCR mixtures and reactions followed by melting curve analysis and agarose gel electrophoresis were performed following Kumar et al. (2013). Three technical replicates for each biological replicate were maintained for qRT-PCR analysis.

## Results

### WRKY transcription factors of *Setaria*

HMM search for WRKY proteins in *Setaria italica* showed the presence of 113 WRKY proteins (SiWRKY), which was in agreement with the numbers reported in Plant Transcription Factor Database v3.0 (Jin et al., 2014) and Foxtail millet Transcription Factor Database (Bonthala et al., 2014). Among these, four SiWRKY proteins (Si031469 m, Si030012 m, Si029764 m, and Si036581 m) were found to be the products of alternate transcripts. In case of *S. viridis*, 50 WRKY TF sequences were identified (SvWRKY). Domain analysis of both SiWRKY and SvWRKY proteins using HMMSCAN and ScanProsite web tools revealed that four SiWRKY and six SvWRKY proteins did not possess the consensus WRKY DNA-binding domain (PF03106). The resultant 105 SiWRKY and 44 SvWRKY sequences (Supplementary Table S2) were used in further studies. Among the 105 SiWRKY proteins, SiWRKY099 was identified to be the smallest protein with 93 amino acids (aa), whereas the largest one was SiWRKY011 (1290 aa). The molecular weights of the proteins also varied according to protein size ranging from 10.3 kDa (SiWRKY099) to 145.8 kDa (SiWRKY011). In case of SvWRKY, the smallest proteins were SvWRKY008 (204 aa) and SvWRKY025 (207 aa), while the largest protein was SvWRKY031 (1290 aa). The molecular weight of SvWRKY proteins ranged from 21.6 kDa (SvWRKY008) to 145.8238 kDa (SvWRKY031). Isoelectric point (pI) of SiWRKY and SvWRKY proteins ranged from 4.8 (SiWRKY056) to 10.1 (SiWRKY037) and 5 (SvWRKY026) to 11.8 (SvWRKY006), respectively. The large variation in protein features might denote the presence of putative novel variants. Instability index of these proteins showed that most proteins (99 SiWRKY and 41 SvWRKY) were unstable (Supplementary Table S2).

### Classification of SiWRKY and SvWRKY proteins

WRKY proteins are classified into three major groups (I, II, and III) based on the conserved WRKY domain and zinc finger-like structure (Rushton et al., 1995). Group I has two WRKY domains as well as CX_4−5_CX_22−23_HXH structure, group II has one WRKY domain with conserved zinc-finger motif sequence, whereas group III has one WRKY domain and CX_4−5_CX_22−23_HXC structure (Eulgem et al., 2000). Group II proteins are further classified into five sub-groups (IIa–IIe) based on the conservation of amino acid motifs outside the WRKY domain (Park et al., 2005). Sequence alignment of SiWRKY and SvWRKY showed that all proteins, except SiWRKY044, SiWRKY063, SvWRKY005, SvWRKY007, SvWRKY008, and SvWRKY011, possess conserved WRKY domain and zinc finger-like structure. These exceptional WRKY proteins were classified as group IV. However, these proteins could represent pseudogenes or sequencing and assembly errors (Xie et al., 2005; Ross et al., 2007).

**Figure 1:**
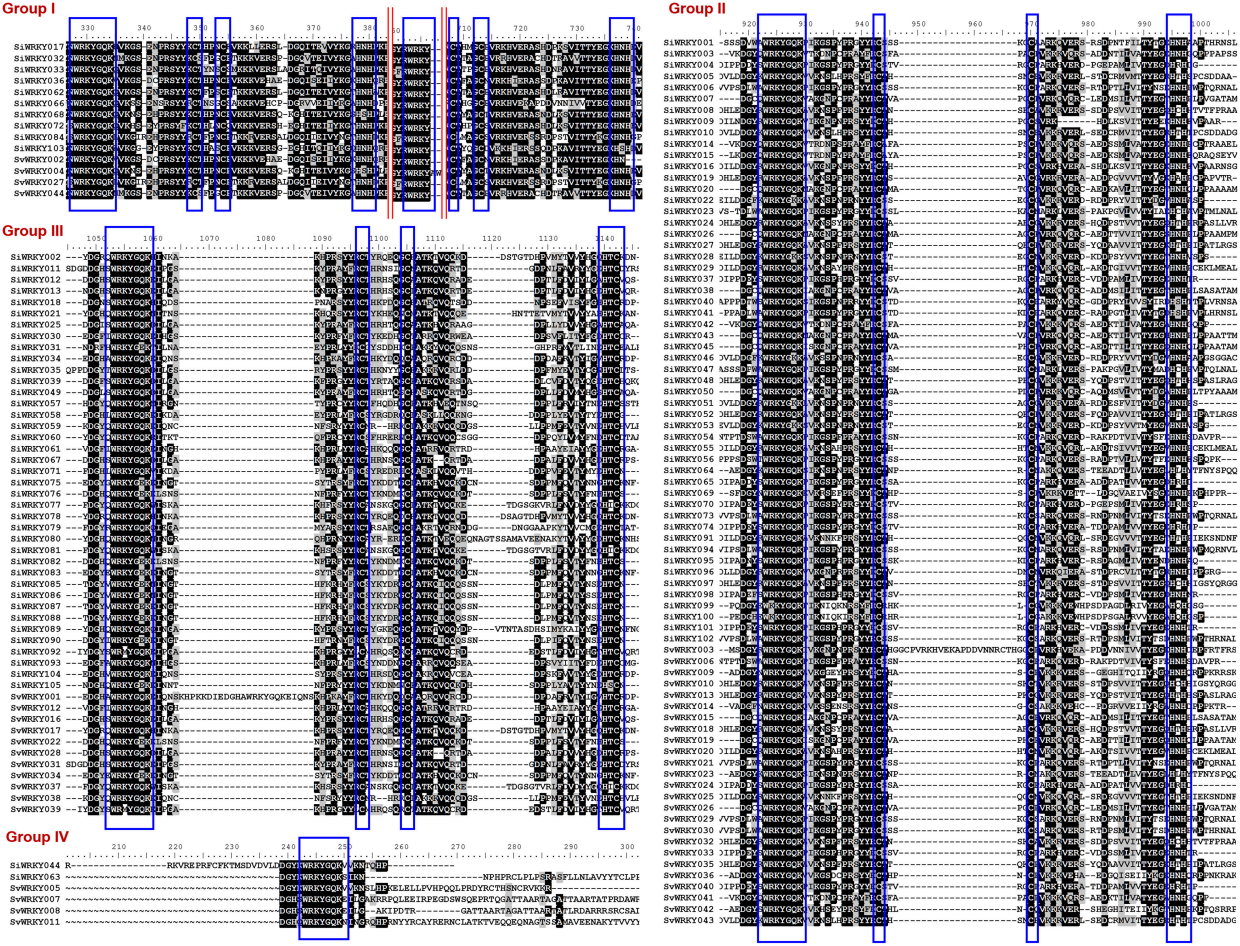
**Multiple sequence alignment of three major groups of SiWRKY and SvWRKY proteins**. Group I, II, III, and IV proteins have been aligned separately and the consensus motifs are highlighted in blue boxes. Highly conserved amino acids have been shown in black boxes, less conserved in gray boxes, while amino acids with no similarly were indicated in black texts. The red lines in group I proteins indicate that the intermittent sequences, which were less conserved were not shown.

**Figure 2:**
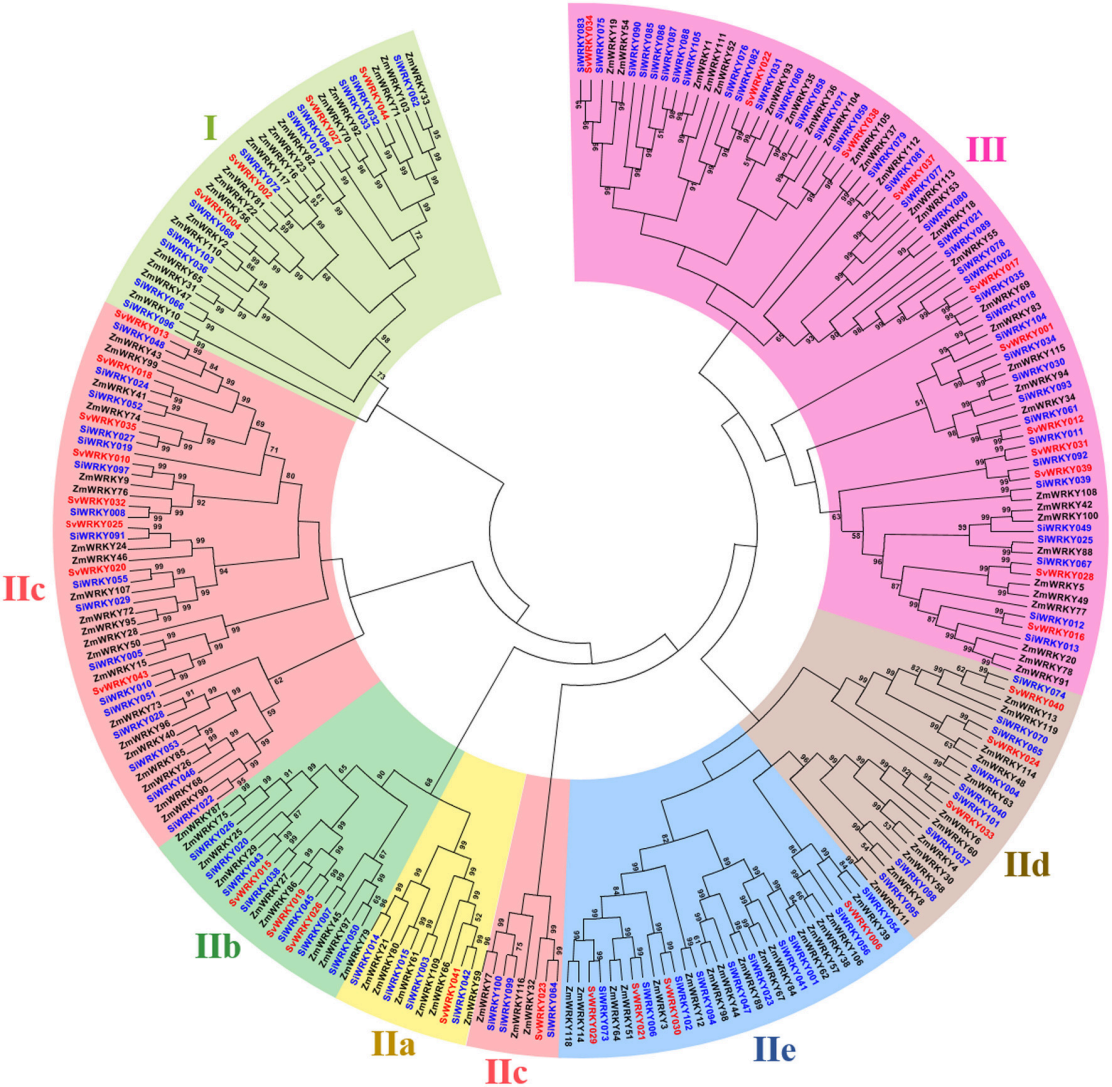
**Unrooted Neighbor-Joining tree constructed with WRKY proteins of ***Setaria italica*** (SiWRKY), ***S. viridis*** (SvWRKY), and ***Zea mays*** (ZmWRKY)**. SiWRKY and SvWRKY protein IDs are highlighted in blue color and groups are differentiated with different colors.

Among the remaining 103 SiWRKY proteins, 10 belong to group I, 54 to group II and 39 to group III, whereas in case of SvWRKY proteins, 6 belong to group I, 23 to group II and 11 to group III (Figure 1). The first WRKY domain of group I proteins possesses a conserved WRKYGQK amino acid motif, whereas the second domain lacked the GQK signature. Both the WRKY domains were followed by conserved CX_4_CX_22−23_HXH structure. Interestingly, SvWRKY004 was observed to possess three WRKY domains followed by zinc finger-like structures. In case of group IV proteins, the conserved WRKYGQK domain was present in the N-terminal region (Figure 1). Phylogenetic analysis of group I, II, and III proteins of SiWRKY, SvWRKY, and ZmWKRY (Wei et al., 2012) confirmed the group-wise classification and also enabled the sub-classification of group II proteins (Figure 2). Group IV proteins deduced through sequence alignment were not included in phylogenetic analysis as they represent the products of pseudogenes or sequencing and assembly errors (Xie et al., 2005; Ross et al., 2007; Wei et al., 2012). Among the 54 group II SiWRKY proteins, 5 belong to IIa, 8 to IIb, 20 to IIc, 9 to IId, and 12 to IIe. Similarly, two SvWRKY proteins belong to group IIa, 3 to IIb, 9 to IIc, 4 to IId, and 5 to IIe. Interestingly, group IIc was interrupted by the members of IIb and IIa (Figure 2). A similar observation was reported by Wei et al. (2012) in maize, wherein the phylogenetic tree of WRKY proteins from *Arabidopsis*, maize, rice, barley, and *Physcomitrella patens* showed the interruption in group IIc. Domain analysis using HMMSCAN and PROSITE tools revealed the presence of additional NB-ARC domain (PF00931) in SiWRKY011 and SvWRKY031, and domain of unknown function (PF12204) in SiWRKY011 and SiWRKY096 (Supplementary Table S3).

### Structure, location, and duplication of *SiWRKY* genes

**Figure 3:**
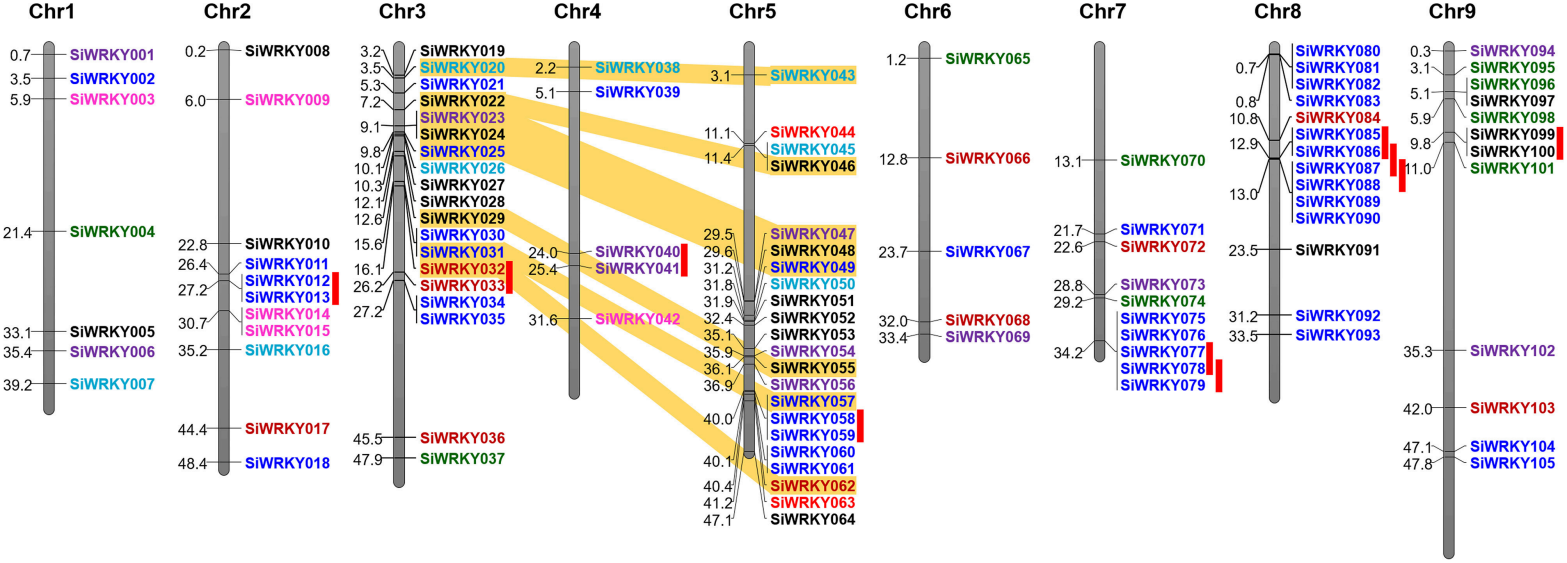
**Physical map of ***SiWRKY*** genes showing their chromosomal locations**. Vertical bars represent the chromosomes and numbers at the left indicate the position of genes (in Mb). Tandemly duplicated gene pairs are indicated with red vertical bars and segmentally duplicated gene pairs are connected with yellow bands. Colors of *SiWRKY* IDs correspond to the different groups.

Positions of introns and exons within the *SiWRKY* genes and their chromosomal locations were determined. However, this could not be performed for *SvWRKY* genes since the genome sequence data of *S. viridis* is not released in public database, till date. Gene structure prediction showed the numbers and arrangement of introns and exons within the *SiWRKY* genes (Supplementary Figure S1). The majority of *SiWRKY* genes (59; ~56%) were found to contain two introns, whereas 22 genes (~21%) have a single intron. Thirteen *SiWRKY* genes (~12%) have three introns, while 5 (~5%) and 4 (~4%) genes have four and five introns, respectively. A maximum of 10 introns were found to be present in *SiWRKY096* and the *SiWRKY065* gene was intronless (Supplementary Figure S1). The length of *SiWRKY* genes was also observed to be variable ranging from 0.6 kb (*SiWRKY019*) to 7.5 kb (*SiWRKY103*). Physical mapping of all the 105 *SiWRKY* genes onto nine chromosomes of *S. italica* revealed an uneven distribution of these genes in the genome (Figure 3). Among the four groups, members of group II and III were present in all the nine chromosomes, whereas group I *SiWRKY* genes were not present in chromosomes 1 and 4. Two members of group IV, namely *SiWRKY044* and *SiWRKY063*, were present in chromosome 5. Subsequently, the expansion of WRKY gene family in *S. italica* genome was examined using MCScanX tool, which showed that 10 and 8 *SiWRKY* gene pairs underwent tandem and segmental duplications, respectively (Figure 3). The tandemly duplicated genes include one pair of group I (in chromosome 3), two pairs of group II (in chromosomes 4 and 9), and seven pairs of group III genes (in chromosomes 1, 5, 7, and 8). Segmental duplication was found to occur between the *SiWRKY* genes of chromosome 3 and 5, and not in other chromosomes (Figure 3).

### Gene ontology annotation and analysis of cis-acting elements

**Figure 4:**
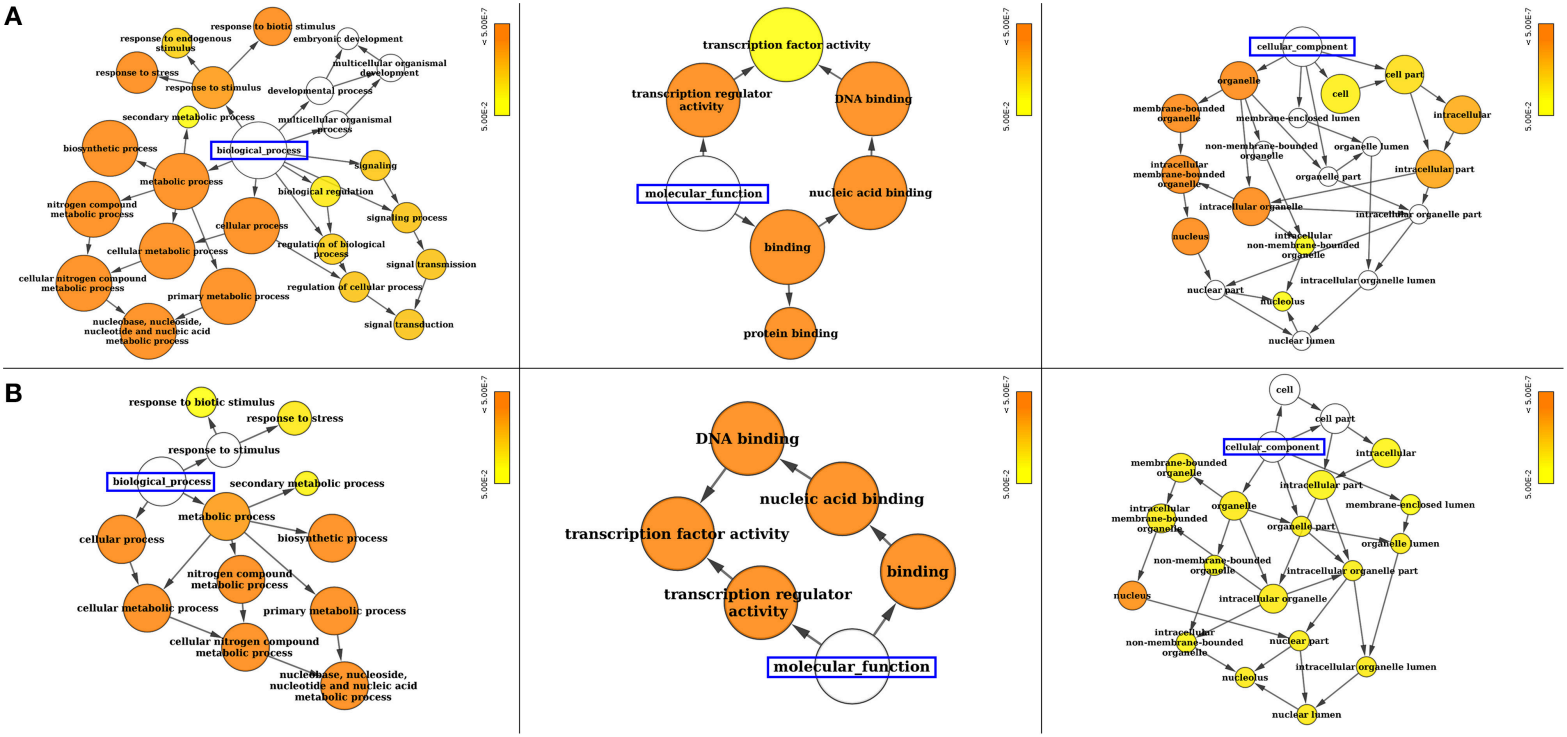
**Gene ontology (GO) enrichment analysis of (A) ***SiWRKY*** and (B) ***SvWRKY*** genes**. The number of genes falling in each GO category is directly proportional to the node size. The nodes are color shaded according to the significance level (corrected *P*-value).

Gene ontology (GO) annotation of SiWRKY and SvWRKY proteins was performed using Blast2GO and Cytoscape tools and showed the involvement of these proteins in different biological processes and molecular functions (Supplementary Table S4). A majority of these proteins were predicted to be involved in response to stress as well as cellular, metabolic and biosynthetic processes (biological process; *P* ≤ 2.2 × 10^−6^) (Figure 4). The molecular functions of these proteins corresponded to transcription regulator activity (*P* ≤ 4.2 × 10^−13^). Further, cellular component analysis revealed the localization of these gene products in nucleus (Figure 4). Promoter analysis of *SiWRKY* genes showed the presence of 284 cis-regulatory elements (CREs), of which some elements were present in all the 105 genes, whereas a few were unique to one or two genes of the entire family (Supplementary Table S5). ARR1AT (element involved in cytokinin responsiveness), CAATBOX1 (element in enhancer regions of the promoter), CACTFTPPCA1 (element involved in mesophyll-specific gene expression of C_4_ phosphoenolpyruvate carboxylase gene in C_4_ plants), DOFCOREZM (target binding site of Dof proteins), EBOXBNNAPA (target binding site of bHLH and MYB-transcription factor), GATABOX (light responsive element), MYCCONSENSUSAT (MYC recognition site), and WRKY71OS (binding site of WRKY TFs) were present in the upstream region of all *SiWRKY* genes. In contrast, few CREs were found to be present in only one *SiWRKY* gene (Supplementary Table S5). This includes ABADESI2 (Synthetic element related to response to abscisic acid and to desiccation; in *SiWRKY009*), ABRE2HVA1 (ABA responsive element; in *SiWRKY053*), ACGTSEED3 (bZIP transcription activator binding site; in *SiWRKY015*), AGL2ATCONSENSUS (MADS binding site; in *SiWRKY034*), AUXRETGA2GMGH3 (auxin responsive element; in *SiWRKY017*), EREGCC (ethylene responsive element; in *SiWRKY051*), HSE (heat shock responsive element; in *SiWRKY047*), MREATCHS (MYB Recognition Element; in *SiWRKY094*), POLLEN2LELAT52 (required for pollen specific expression; *SiWRKY002*), and S2FSORPL21 (leaf-specific, light-independent regulatory element; in *SiWRKY094*; Supplementary Table S5).

### Comparative mapping in related grass genomes and Ks dating of paralogs and orthologs

**Figure 5:**
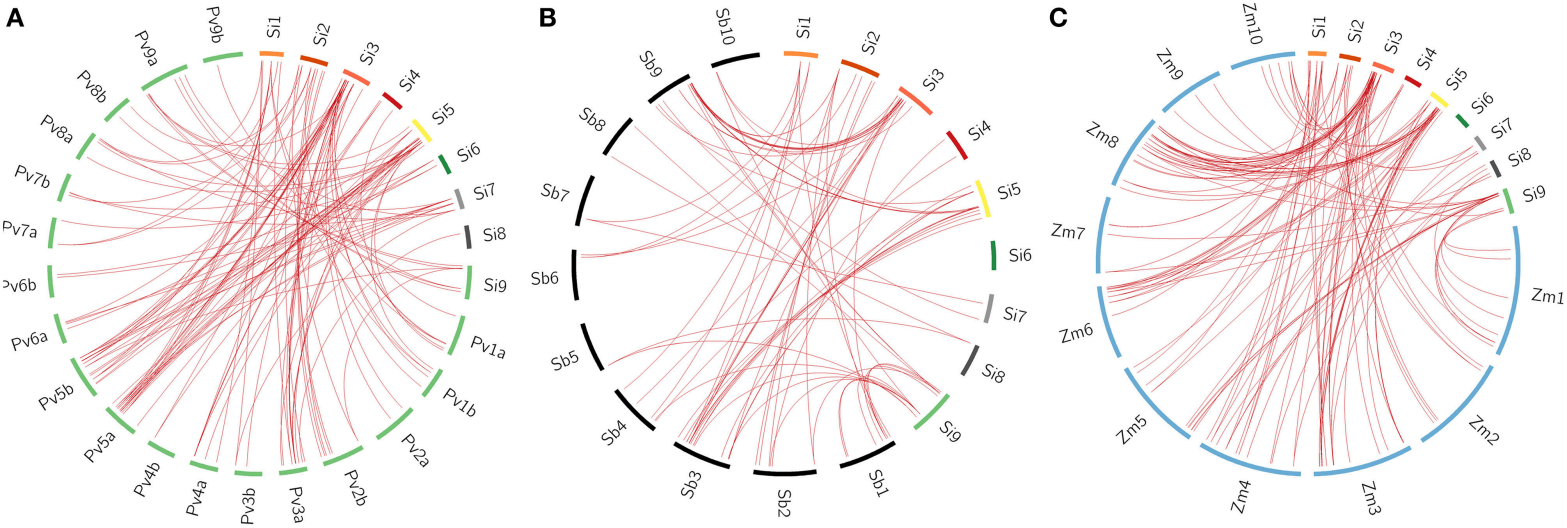
**Synteny map showing the orthologous gene positions of ***WRKY*** genes in ***Setaria italica*** (Si) and (A) ***Panicum virgatum*** (Pv), (B) ***Sorghum bicolor*** (Sb), (C) ***Zea mays*** (Zm)**. Each block represents individual chromosome and the orthologous genomic regions are marked with red lines.

All the 105 *SiWRKY* genes and 44 SvWRKY proteins were subjected to BLAST search against the database of switchgrass (*Panicum virgatum*), sorghum (*Sorghum bicolor*) and maize (*Zea mays*) to identify corresponding orthologs (>90% similarity). Potential orthologs were confirmed by reciprocal BLAST (Figure 5; Supplementary Tables S6–S11). A total of 60 *SiWRKY* genes (~57%) showed syntenic relationship with maize (Supplementary Table S8), followed by switchgrass (~54%; Supplementary Table S6) and sorghum (~40%) (Supplementary Table S7). In case of SvWRKY proteins, maximum synteny was observed with switchgrass (31, ~70%; Supplementary Table S9), followed by maize (24, ~55%; Supplementary Table S11) and sorghum (20, ~45%; Supplementary Table S10). Further, the effect of Darwinian positive selection in duplication and divergence of *WRKY* genes was examined by estimating the ratios of non-synonymous (Ka) vs. synonymous (Ks) substitution for paralogous as well as orthologous gene pairs. The Ka/Ks ratio for tandemly duplicated gene pairs ranged from 0.09 to 0.18 with an average of 0.13 (Supplementary Table S12), while for segmentally duplicated gene pairs, the ratio ranged from 0.06 to 0.14 with an average of 0.1 (Supplementary Table S13). Both the tandem and segmental duplications have been estimated to occur around 29 million years ago (mya) and 23 mya, respectively (Supplementary Tables S12, S13). Similarly, the average Ka/Ks ratios of orthologous gene pairs of *S. italica* - *P. virgatum, S. italica* - *S. bicolor*, and *S. italica* - *Z. mays* were estimated as 0.94, 0.19, and 0.19, respectively (Supplementary Tables S6–S8). In case of *S. viridis* - *P. virgatum, S. viridis* - *S. bicolor*, and *S. viridis* - *Z. mays* orthologs, the Ka/Ks ratios were 0.79, 0.21, and 0.18, respectively (Supplementary Tables S9–S11). This revealed that the orthologous gene pairs underwent natural selection (Ka/Ks < 1). The estimated time of divergence of *S. italica* and *P. virgatum* was 4.7 mya, whereas *S. italica* and *S. bicolor* as well as *Z. mays* diverged around 27 mya. Similar estimates were observed in case of *S. viridis* - *P. virgatum* (4.7 mya), *S. viridis* - *S. bicolor* (26.8 mya), and *S. viridis* - *Z. mays* (27.8 mya) orthologs.

### *In silico* expression profiling of *SiWRKY* and *SvWRKY* genes

**Figure 6:**
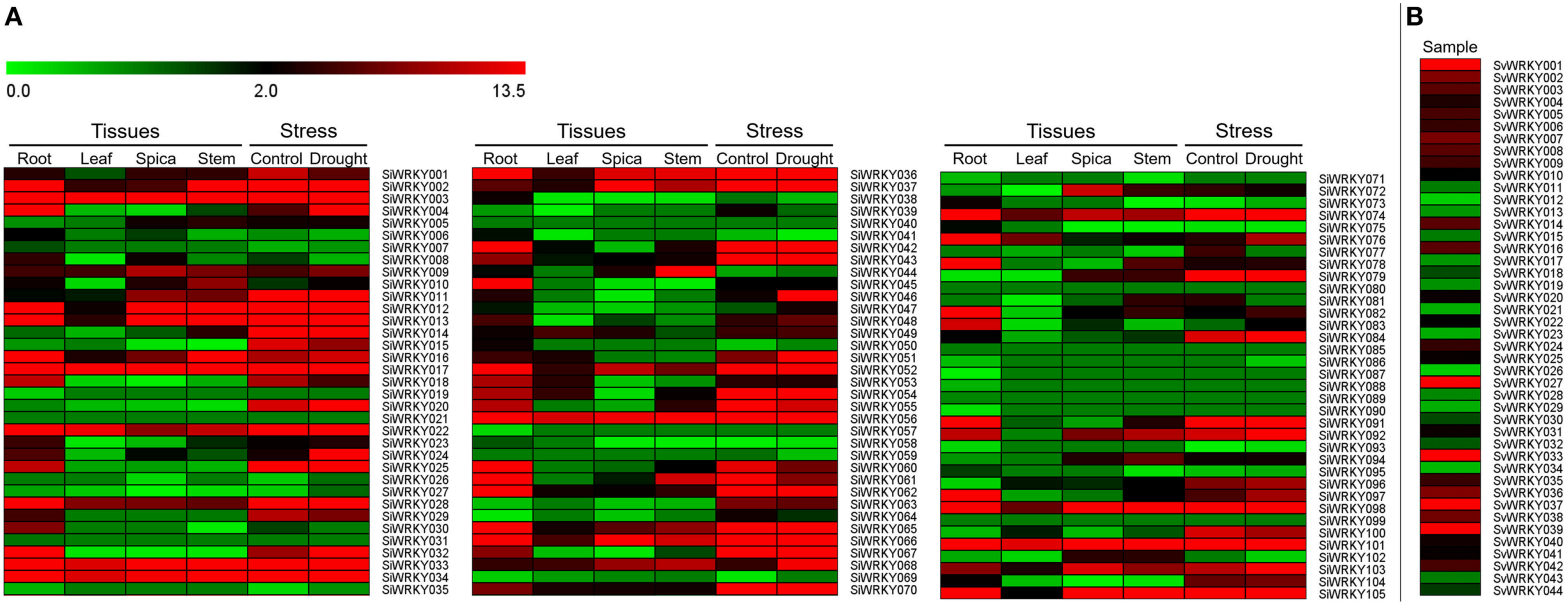
**Heatmap showing the expression pattern of (A) ***SiWRKY*** genes in four tissues, namely root, leaf, spica, stem, and dehydration stress library of ***Setaria italica***, and (B) ***SvWRKY*** genes in pooled transcriptome of ***S. viridis*** samples across three developmental stages, namely seed germination, vegetative growth, and reproduction in different tissues including leaf, stem, node, crown, root, spikelet, floret, and seed tissues**. The colored bar at top left represents relative expression value, where 0.0, 2.0, and 13.5 denotes low, medium, and high expression, respectively.

Expression pattern of *SiWRKY* genes in four tissues, namely root, leaf, spica, and stem revealed a differential expression pattern (Figure 6). A few genes including *SiWRKY003, SiWRKY017, SiWRKY033, SiWRKY034, SiWRKY056*, and *SiWRKY101* were found to be highly expressed in all the tissues. Tissue-specific higher expression of *SiWRKY028, SiWRKY032, SiWRKY042, SiWRKY045, SiWRKY060, SiWRKY062, SiWRKY078*, and *SiWRKY091* in root and *SiWRKY044* in stem was also observed. Some genes such as *SiWRKY006, SiWRKY019, SiWRKY026, SiWRKY039, SiWRKY057*, etc. did not show any expression in all the four tissues (Figure 6). In case of expression profiles in dehydration stress library, relatively higher expression of *SiWRKY004, SiWRKY024, SiWRKY046*, and *SiWRKY068* was observed in stressed sample as compared to control. Downregulation of a few genes viz., *SiWRKY60, SiWRKY61*, etc. was also seen (Figure 6). Expression patterns of *SvWRKY* genes in pooled RNA isolated from samples across three developmental stages, namely seed germination, vegetative growth, and reproduction in different tissues including leaf, stem, node, crown, root, spikelet, floret, and seed tissues showed higher transcript abundance of *SvWRKY001, SvWRKY027, SvWRKY033, SvWRKY037*, and *SvWRKY039*. However, the majority of *SvWRKY* genes showed no or negligible expression (Figure 6).

### Expression pattern of *SiWRKY* genes in response to stress and hormone treatments

**Figure 7:**
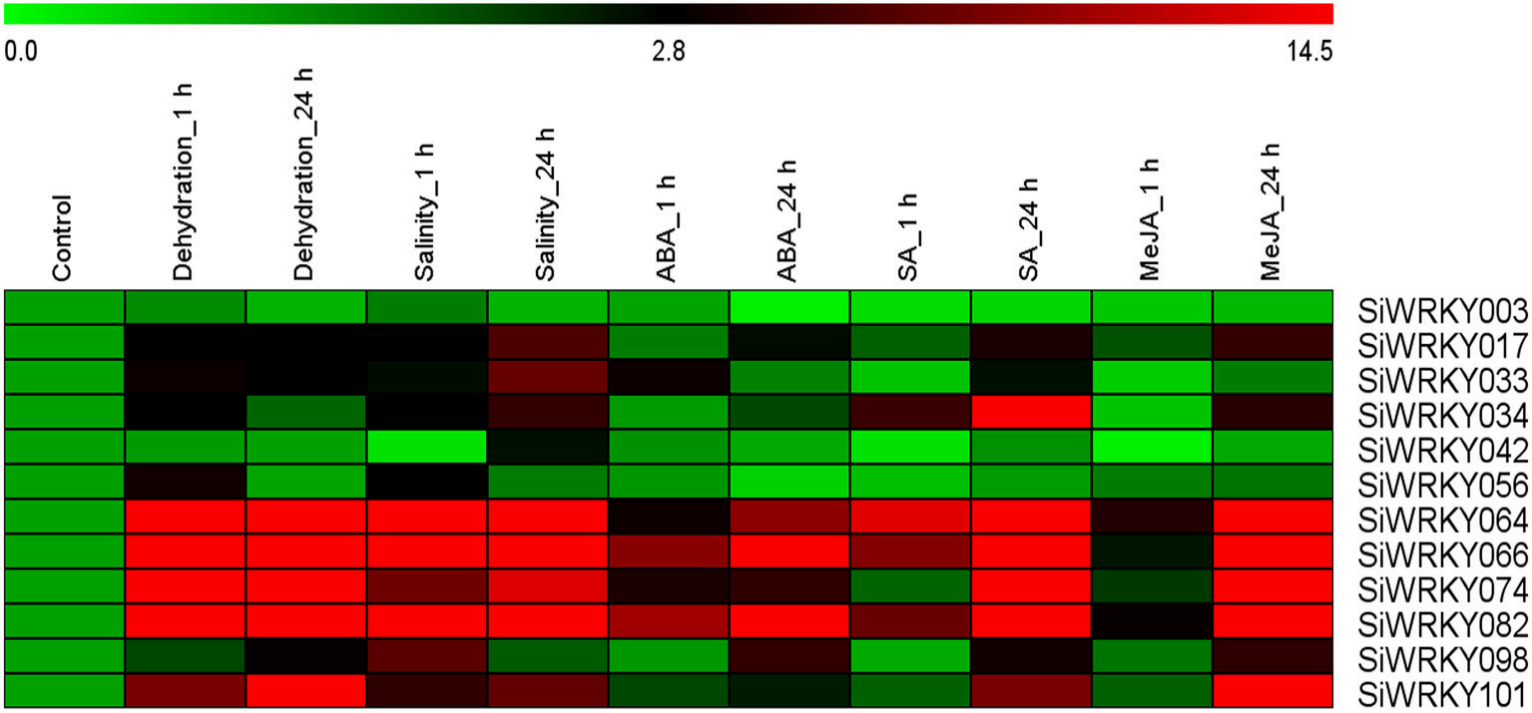
**Relative transcript levels of candidate ***SiWRKY*** genes in response to treatment with stresses and hormones**. Expression profiles of *SiWRKY* genes in *Setaria italica* seedlings exposed to dehydration, salinity, abscisic acid (ABA), salicylic acid (SA), and methyl jasmonate (MeJA) for two time points (1 h and 24 h) were analyzed by qRT-PCR and presented as heatmap. The scale bar at the top represents relative expression value. Relative fold expression values are presented as bar diagram in Supplementary Figure S2.

To investigate the expression of *SiWRKY* genes in response to abiotic stress and hormone treatments, 12 genes were selected based on their differential expression pattern in RNA-seq libraries of four tissues and under drought stress (Figure 6). Additionally, the genes were resourced from the nine chromosomes of *S. italica* in order to provide a genome-wide coverage (Figure 2). The expression profiles of the 12 candidate genes were examined during early (1 h) and late (24 h) stages of dehydration, salinity, ABA, SA, and MeJA treatments. The relative transcript abundance assessed through qRT-PCR showed a differential expression pattern of all the *SiWRKY* genes (Figure 7). Few genes including *SiWRKY003, SiWRKY017, SiWRKY033, SiWRKY042*, and *SiWRKY056* did not show any significant expression throughout the experiments, whereas *SiWRKY034* was highly expressed only during the late phase of SA treatment. During dehydration and salinity stress, *SiWRKY064, SiWRKY066, SiWRKY074*, and *SiWRKY082* were found to be upregulated at both the time points, in which, significant upregulation of *SiWRKY064* and *SiWRKY082* at late phase of salinity stress, and *SiWRKY066* and *SiWRKY074* at both the phases of dehydration were observed (Supplementary Figure S2). In case of hormone treatments, all these four genes were found to be highly expressed during late phase. In addition to these, *SiWRKY101* was observed to be upregulated during late phase of dehydration and MeJA treatment. The fold expression of *SiWRKY064* and *SiWRKY082* were significantly higher during both the phases of stress and at late phase of hormone treatments, suggesting their potential as candidates for functional characterization.

## Discussion

WRKY transcription factors have been reported to play multiple roles in regulating normal growth and development, and in response to environmental stimuli in plants (Rushton et al., 2010). This class of TFs are one of the well-studied proteins whose mechanism of action, autoregulation and cross-regulation in signaling and evolution have been reported (Bakshi and Oelmüller, 2014). Though initially considered as vital players of biotic stress tolerance, WRKY TFs were later discovered to play significant roles in conferring tolerance to diverse abiotic stresses including salinity (Jiang and Yu, 2009; Chen et al., 2010), drought, heat (Li et al., 2009, 2011), cold (Zou et al., 2010), H_2_O_2_(Song et al., 2009), ozone oxidative stress (Jiang and Deyholos, 2009), UV radiation (Jiang and Deyholos, 2009), sugar starvation (Song et al., 2010), phosphate depreviation (Chen et al., 2009) and wounding (Shang et al., 2010). Further, numerous reports have indicated the response of a single WRKY gene to several stress factors, thus highlighting the diverse regulatory role of WRKY proteins in stress response (Wei et al., 2008; Jiang and Deyholos, 2009; Li et al., 2009, 2011; Chen et al., 2012). The expression of WRKY TFs in response to broad-spectrum abiotic stresses suggests their participation in regulation of signaling mechanisms associated with transcriptional reprogramming during environmental stress. Genome-wide identification of WRKY TFs has been performed in many crop plants and their expression profiling in response to various abiotic stresses have been studied.

Recently, C_4_ crops are gaining momentum in stress biology research owing to their improved water-use efficiency and nitrogen-use efficiency (Sadras et al., 2011). C_4_ photosynthesis also confers tolerance to crops against abiotic stress, particularly to drought and heat (Sadras et al., 2011). *Setaria italica* and its wild progenitor *S. viridis*, have recently been identified as model crops for studying C_4_ photosynthesis and abiotic stress tolerance due to their small genome, short life span, inbreeding nature and ability to withstand adverse environmental conditions (Brutnell et al., 2010; Wang et al., 2011; Diao et al., 2014; Muthamilarasan and Prasad, 2015). Furthermore, both the crops share maximum genetic synteny with various biofuel grasses such as switchgrass, napiergrass and pearl millet and therefore, *S. italica* and *S. viridis* have also been regarded as model systems for bioenergy research (Li and Brutnell, 2011; Lata et al., 2013; Brutnell et al., 2015; Muthamilarasan and Prasad, 2015) and nutrition studies (Muthamilarasan et al., in press). Therefore, in view of the importance of *S. italica* and *S. viridis* in abiotic stress biology, the present investigation was performed to identify and characterize WRKY TFs using computational tools and examine their expression patterns in response to abiotic stress and hormone treatments.

In this study, 105 *WRKY* genes from *S. italica* genome (*SiWRKY*) and 44 from *S. viridis* transcriptome (*SvWRKY*) were identified. Comparison of the number of *WRKY* genes in *S. italica* with other sequenced grass genomes namely maize (163 genes), sorghum (110 genes) and rice (*O. sativa* subsp. *indica*; 109 genes) has shown that *S. italica* has comparatively lesser number of genes. However, *Brachypodium* has a minimum of 87 genes, owing to its smaller genome size. Similar comparisons of the number of *WRKY* genes among all the sequenced plants showed that soybean has the maximum number of *WRKY* genes (233), followed by cotton (219), whereas the primitive plants of Chlorophyta have one to two genes. Interestingly, the genome of *Physcomitrella patens* has 41 *WRKY* genes. Only 44 WRKY proteins were identified from the transcriptome of *S. viridis* due to the non-availability of genome sequence information and this number is expected to increase when the whole genome sequence is released in public domain. Examining the protein properties of SiWRKY and SvWRKY TFs revealed large differences in amino acid length, molecular weight and isoelectric point of these proteins, and these variations could be attributed to the presence of putative novel variants, which needs to be validated.

Sequence alignment and phylogenetic analysis of SiWRKY and SvWRKY proteins classified them into three major groups (I, II, and III) based on the WRKY domain and conserved zinc finger-like motif. In addition, a distinct class of WRKY proteins classified as group IV has been identified with two members of SiWRKY and four members of SvWRKY. These proteins possess only the WRKY domain and not the zinc finger-like motif. Sequence alignment and phylogenetic analysis showed that a majority of SiWRKY proteins belong to group II (54) followed by group III (39) and group I (10). Similar case was observed in SvWRKY proteins, where a maximum of 23 proteins belong to group II, 11 to group III, and 6 to group I. This is in agreement with the distribution reported in maize (Wei et al., 2012). The position of WRKY domain and associated zinc finger-like structures in SiWRKY and SvWRKY was investigated through multiple sequence alignment and domain analyses tools, namely HMMSCAN and ScanProsite. The analyses revealed that the distribution of phylogenetic groups corresponds well with the domain structures and sequence conservation. It showed three interesting observations: (i) two proteins of SiWRKY (SiWRKY044 and SiWRKY063) and four SvWRKY proteins (SvWRKY005, SvWRKY007, SvWRKY008, and SvWRKY011) possess only WRKY domain and lack zinc finger-like structure. (ii) two additional domains, namely NB-ARC and DUF were present in SiWRKY011 and SvWRKY031, and SiWRKY011 and SiWRKY096, respectively. and (iii) SvWRKY004 has three WRKY domains followed by zinc finger-like structures.

Physical mapping of *SiWRKY* genes on the nine chromosomes of *S. italica* showed that maximum number of genes were present on chromosomes 5 (22 genes; ~21%) and 3 (19 genes; ~18%), and a minimum of 5 genes each (~5%) were present on chromosomes 4 and 6. The maximum number of genes on chromosomes 5 and 3 could be attributed to the occurrence of segmental duplication, as revealed by MCScanX analysis. Eight genes in these chromosomes were segmentally duplicated, and in addition, 10 gene pairs were identified to be tandem duplicates. The Ks dating and estimation of Ka/Ks ratios of duplicated gene pairs showed that these genes underwent intense purifying selection. The time of duplication of tandemly and segmentally duplicated gene pairs were estimated as ~26 and ~23 million years ago (mya), which were in congruence with the whole genome tandem and segmental duplication reported to have occurred around 25–27 and 18–22 mya (Zhang et al., 2012). This also demonstrates the effect of chromosomal duplication events in shaping the distribution and organization of *WRKY* genes in *S. italica* genome.

Comparative mapping of *SiWRKY* genes and SvWRKY proteins on the switchgrass, sorghum and maize databases was performed to understand the orthologous relationships between the grass genomes. *SiWRKY* genes showed maximum synteny with maize (~57%), followed by switchgrass (~54%) and sorghum (~40%), whereas SvWRKY proteins showed maximum orthology with switchgrass (~70%), followed by maize (~55%) and sorghum (~45%). Though higher percentage of orthology was expected between *Setaria* and switchgrass owing to their extensive gene-level synteny, *SiWRKY* genes were found to be more homologous to maize. However, SvWRKY revealed the syntenic pattern with respect to decrease in synteny with increase in phylogenetic distance, between these crops. Estimation of time of divergence of orthologous gene pairs revealed that *S. italica* and switchgrass *WRKY* genes diverged around 4.7 mya, whereas divergence between *S. italica WRKY* genes and those of maize and sorghum occurred around 27 and 27.5 mya, respectively. Similarly, *S. viridis* and switchgrass *WRKY* genes were predicted to have diverged around 4.7 mya, while *S. viridis* and maize, and sorghum *WRKY* genes diverged around 26.8 and 27.8 mya, respectively. These findings are in accordance with the period of divergence of Poaceae members as reported by Zhang et al. (2012). The comparative map constructed using orthologous *WRKY* genes demonstrated the frequent occurrence of nested chromosomal fusions in the grass genomes. Further, this comparative map would be useful in choosing candidate *WRKY* genes from these genomes for functional characterization.

The publicly available transcriptome data of four different tissues and dehydration stress library of *S. italica*, and pooled tissue library of *S. viridis* were processed using in-house perl scripts and computational tools to derive the RPKM expression values for *SiWRKY* and *SvWRKY* genes. The heatmap generated using these expression values showed tissue-specific and condition-specific expression patterns of *WRKY* genes. Relatively higher expression of few genes in all the tissues, or in any one tissue or only during dehydration stress suggested the multifaceted roles of *WRKY* genes in diverse molecular and physiological activities. This data could be exploited for selecting candidate genes showing distinct expression pattern for delineating their functional roles. Based on this heatmap and physical map data, twelve candidate *SiWRKY* genes were chosen for expression profiling under different abiotic stress (dehydration and salinity) and hormone (ABA, SA, and MeJA) treatments (at two time points). These genes showed differential expression pattern in the four tissues (root, stem, leaf, and spica) and drought stress library as deduced using RNA-seq expression data. Further, the genes were also chosen to represent all the nine chromosomes of foxtail millet, to provide a representative genome-wide coverage. The qRT-PCR analysis of these genes showed their differential expression patterns during exposure to stresses and hormones, and this suggested the putative involvement of *WRKY* genes in stress response mechanism and their regulation in response to phytohormones. Overall, the qRT-PCR analysis revealed that *SiWRKY066* and *SiWRKY082* could be potential candidates for further functional characterization and for delineating their roles in abiotic stress signaling.

## Conclusions

With the advancement of high-throughput technologies and strategies, including physiology, chemical genetics, and computational approaches, the role of WRKY TFs in signal transduction and gene regulation has been well studied in all the major crops and tree species. However, no such study on WRKY TFs has been conducted in *S. italica* and *S. viridis*, which are now considered as model systems for investigating C_4_ photosynthesis, biofuel traits and abiotic stress tolerance mechanisms. Considering the importance of these crops and WRKY TFs, the present study used comprehensive computational approaches to identify and characterize WRKY gene family members. The identified members were used for construction of a physical map, duplication studies, phylogenetic analysis, gene ontology annotation, promoter analysis, comparative mapping, and evolutionary studies. In addition, *in silico* expression profiling of *SiWRKY* and *SvWRKY* genes were performed to understand the expression pattern of these genes in different tissues and dehydration stress conditions. Expression profiling of candidate *SiWRKY* genes under abiotic stress and hormone treatments showed differential expression pattern of these genes, thus providing an indication of their regulatory functions under stress conditions.

## Author contributions

MP conceived and designed the experiments. MM, VB, RK, JJ, SS, KN performed the experiments. MM analyzed the results and wrote the manuscript. MP approved the final version of the manuscript.

## Funding

Research on foxtail millet genomics at MP's laboratory is funded by the Core Grant of National Institute of Plant Genome Research, New Delhi, India.

### Conflict of interest statement

The authors declare that the research was conducted in the absence of any commercial or financial relationships that could be construed as a potential conflict of interest.
