# Supplementary material for: Global analysis of WRKY transcription factor superfamily in Setaria identifies potential candidates involved in abiotic stress signaling
Source: Front Plant Sci. 2015 Oct 26;6:910. doi: 10.3389/fpls.2015.00910 (PMC4654423; doi:10.3389/fpls.2015.00910)
Supplement: Supplementary file 1 [file Table1.DOC]

**Supplementary Table S1.** Details of primers used in qRT-PCR analysis.

| **Gene** | **Forward primer (5'-3')** | **Reverse primer (5'-3')** |
| --- | --- | --- |
| *SiWRKY003* | CGAGAACAATGGACACGG | GCCGTGATGATGTTCTTCC |
| *SiWRKY017* | AGGTCGTTTGCTGTTTGGAT | GCTCTTTGGTTCTATCCC |
| *SiWRKY033* | ATTTGTTTATTCGTTGGATG | GCTACAAAGACACAGGACCGT |
| *SiWRKY034* | AATAATGGCTGCGTGGAT | TTGTTGAAGTGACGCTGA |
| *SiWRKY042* | GACGAAGCCTGGATTAGC | ATTCTACAGTCTACATACAGC |
| *SiWRKY056* | TACCTTTCACCCTTCCCTTTG | GAATCACATCGCAAACCACTC |
| *SiWRKY064* | GTGACATCTTAGCAATCCCT | TTTCCACCAACTATGTCA |
| *SiWRKY066* | AAGCAACGAAGAGACCAGATG | CAAATACCAGACAATGCG |
| *SiWRKY074* | CTGATGTCCGTGTAAAAATG | AGAGAACAGGAGATAACGGA |
| *SiWRKY082* | GCGTCAAGTGCTGGGAGG | TAAGTAAGCAACAAGGCAGTAA |
| *SiWRKY098* | AATGGCGAAAGAAGGAAACT | ACACCATCCCCAGACATACAAC |
| *SiWRKY101* | TGCTGTTTTGTTGTTTGG | CACACTAACCCTGAGCAAC |
| *Actin2* | CGCATATGTGGCTCTTGACT | GGGCACCTAAATCTCTCTGC |
