# Supplementary material for: Global analysis of WRKY transcription factor superfamily in Setaria identifies potential candidates involved in abiotic stress signaling
Source: Front Plant Sci. 2015 Oct 26;6:910. doi: 10.3389/fpls.2015.00910 (PMC4654423; doi:10.3389/fpls.2015.00910)
Supplement: Supplementary file 6 [file Table6.DOC]

**Supplementary Table S6.** The Ka/Ks ratios and estimated divergence time for orthologous *WRKY* genes between *Setaria italica* and *Panicum virgatum*.

| **Foxtail millet WRKY** | **Switchgrass ortholog gene ID** | **% identity** | **Ka** | **Ks** | **Ka/Ks** | **Time of divergence (MYA)** |
| --- | --- | --- | --- | --- | --- | --- |
|
| SiWRKY002 | Pavir.Cb02194 | 91.18 | 0.06 | 0.04 | 1.47 | 3.3 |
| SiWRKY003 | Pavir.Da00323 | 92.02 | 0.01 | 0.05 | 0.19 | 4.1 |
| SiWRKY004 | Pavir.Ea02968 | 100 | 0.05 | 0.06 | 0.89 | 4.4 |
| SiWRKY005 | Pavir.Ga00648 | 90.2 | 0.05 | 0.04 | 1.22 | 3.2 |
| SiWRKY007 | Pavir.Ca01747 | 100 | 0.04 | 0.06 | 0.61 | 4.8 |
| SiWRKY008 | Pavir.Bb00073 | 97.44 | 0.01 | 0.06 | 0.14 | 4.9 |
| SiWRKY009 | Pavir.Hb01804 | 96.67 | 0.01 | 0.08 | 0.14 | 6.4 |
| SiWRKY010 | Pavir.Ga00648 | 97.24 | 0.03 | 0.08 | 0.37 | 6.0 |
| SiWRKY014 | Pavir.Eb03562 | 100 | 0.03 | 0.08 | 0.38 | 5.8 |
| SiWRKY015 | Pavir.Bb02115 | 91.06 | 0.03 | 0.09 | 0.33 | 6.8 |
| SiWRKY016 | Pavir.Bb02418 | 94.17 | 0.02 | 0.04 | 0.40 | 3.3 |
| SiWRKY017 | Pavir.Bb03225 | 90.52 | 0.02 | 0.05 | 0.35 | 3.8 |
| SiWRKY018 | Pavir.Bb03750 | 91.13 | 0.03 | 0.05 | 0.54 | 4.0 |
| SiWRKY019 | Pavir.Eb02690 | 93.58 | 0.02 | 0.05 | 0.38 | 3.5 |
| SiWRKY023 | Pavir.Aa02610 | 96 | 0.02 | 0.06 | 0.26 | 5.0 |
| SiWRKY024 | Pavir.Eb02690 | 91.34 | 0.02 | 0.06 | 0.26 | 4.7 |
| SiWRKY025 | Pavir.Db02095 | 94.12 | 0.02 | 0.07 | 0.22 | 5.5 |
| SiWRKY027 | Pavir.Eb02690 | 94.41 | 0.01 | 0.04 | 0.35 | 3.2 |
| SiWRKY028 | Pavir.Aa00351 | 100 | 0.02 | 0.04 | 0.39 | 3.4 |
| SiWRKY029 | Pavir.Ca01507 | 96.14 | 0.02 | 0.05 | 0.35 | 3.8 |
| SiWRKY030 | Pavir.Ba02544 | 90.2 | 0.09 | 0.09 | 1.03 | 6.7 |
| SiWRKY032 | Pavir.Ia00386 | 91.67 | 0.13 | 0.08 | 1.60 | 6.3 |
| SiWRKY033 | Pavir.Ca01475 | 96.12 | 0.10 | 0.08 | 1.31 | 5.8 |
| SiWRKY034 | Pavir.Cb01173 | 90.91 | 0.07 | 0.07 | 0.94 | 5.4 |
| SiWRKY036 | Pavir.Ca02393 | 93.21 | 0.09 | 0.07 | 1.33 | 5.0 |
| SiWRKY038 | Pavir.Da02223 | 91.44 | 0.09 | 0.05 | 1.67 | 4.0 |
| SiWRKY039 | Pavir.Ea02282 | 94.92 | 0.09 | 0.07 | 1.29 | 5.2 |
| SiWRKY042 | Pavir.Aa03147 | 90.21 | 0.09 | 0.07 | 1.21 | 5.5 |
| SiWRKY043 | Pavir.Ia00516 | 91.74 | 0.08 | 0.08 | 1.12 | 5.8 |
| SiWRKY045 | Pavir.Ea00275 | 91.75 | 0.08 | 0.05 | 1.76 | 3.7 |
| SiWRKY046 | Pavir.Fa01143 | 100 | 0.08 | 0.04 | 1.89 | 3.4 |
| SiWRKY047 | Pavir.Ea02436 | 97.4 | 0.08 | 0.05 | 1.77 | 3.7 |
| SiWRKY048 | Pavir.Eb01898 | 95.77 | 0.13 | 0.04 | 3.03 | 3.2 |
| SiWRKY049 | Pavir.Db02095 | 94.92 | 0.13 | 0.05 | 2.30 | 4.2 |
| SiWRKY051 | Pavir.Eb02454 | 97 | 0.13 | 0.06 | 2.21 | 4.4 |
| SiWRKY052 | Pavir.Ia00386 | 90.62 | 0.13 | 0.06 | 2.05 | 4.7 |
| SiWRKY055 | Pavir.Ca01507 | 93.64 | 0.02 | 0.07 | 0.24 | 5.0 |
| SiWRKY056 | Pavir.Eb01390 | 91.8 | 0.05 | 0.07 | 0.81 | 5.2 |
| SiWRKY057 | Pavir.Ea02967 | 95.65 | 0.05 | 0.07 | 0.81 | 5.2 |
| SiWRKY059 | Pavir.Eb03561 | 95.6 | 0.05 | 0.07 | 0.77 | 5.4 |
| SiWRKY060 | Pavir.Eb03562 | 95.92 | 0.05 | 0.07 | 0.75 | 5.6 |
| SiWRKY062 | Pavir.Ca01475 | 95 | 0.02 | 0.07 | 0.28 | 5.7 |
| SiWRKY064 | Pavir.Ea04136 | 95.9 | 0.13 | 0.07 | 1.84 | 5.4 |
| SiWRKY065 | Pavir.Eb03138 | 97.83 | 0.04 | 0.07 | 0.63 | 5.0 |
| SiWRKY066 | Pavir.Fb01409 | 93.02 | 0.09 | 0.06 | 1.53 | 4.7 |
| SiWRKY070 | Pavir.Hb00911 | 94.42 | 0.06 | 0.06 | 0.99 | 4.4 |
| SiWRKY071 | Pavir.Gb01556 | 91.67 | 0.03 | 0.05 | 0.50 | 4.1 |
| SiWRKY072 | Pavir.Eb03590 | 91.2 | 0.01 | 0.05 | 0.21 | 4.1 |
| SiWRKY076 | Pavir.Ia04238 | 91.11 | 0.10 | 0.06 | 1.56 | 4.9 |
| SiWRKY078 | Pavir.Cb02194 | 90.87 | 0.06 | 0.05 | 1.19 | 3.7 |
| SiWRKY089 | Pavir.Ca00459 | 97.78 | 0.02 | 0.05 | 0.49 | 3.7 |
| SiWRKY094 | Pavir.Ia00085 | 91.07 | 0.05 | 0.05 | 0.99 | 4.1 |
| SiWRKY096 | Pavir.Eb02454 | 91.11 | 0.01 | 0.06 | 0.15 | 4.7 |
| SiWRKY097 | Pavir.Bb00073 | 91.45 | 0.02 | 0.07 | 0.37 | 5.0 |
| SiWRKY098 | Pavir.Ia00625 | 90.35 | 0.02 | 0.05 | 0.44 | 4.2 |
| SiWRKY103 | Pavir.Ia02889 | 94.74 | 0.09 | 0.06 | 1.44 | 5.0 |
| SiWRKY105 | Pavir.Ia03459 | 93.8 | 0.13 | 0.07 | 1.88 | 5.2 |
| **Mean** | | | **0.06** | **0.06** | **0.94** | **4.7** |
