# Supplementary material for: Global analysis of WRKY transcription factor superfamily in Setaria identifies potential candidates involved in abiotic stress signaling
Source: Front Plant Sci. 2015 Oct 26;6:910. doi: 10.3389/fpls.2015.00910 (PMC4654423; doi:10.3389/fpls.2015.00910)
Supplement: Supplementary file 7 [file Table7.DOC]

**Supplementary Table S7.** The Ka/Ks ratios and estimated divergence time for orthologous *WRKY* genes between *Setaria italica* and *Sorghum bicolor*.

| **Foxtail millet WRKY** | **Sorghum ortholog gene ID** | **% identity** | **Ka** | **Ks** | **Ka/Ks** | **Time of divergence (MYA)** |
| --- | --- | --- | --- | --- | --- | --- |
|
| SiWRKY004 | Sobic.003G285500 | 97.37 | 0.04 | 0.19 | 0.20 | 14.6 |
| SiWRKY005 | Sobic.004G298400 | 95.93 | 0.05 | 0.35 | 0.13 | 26.7 |
| SiWRKY008 | Sobic.002G008600 | 95.56 | 0.09 | 0.32 | 0.27 | 24.5 |
| SiWRKY010 | Sobic.006G166300 | 93.6 | 0.03 | 0.19 | 0.15 | 14.3 |
| SiWRKY014 | Sobic.002G202800 | 93.33 | 0.03 | 0.41 | 0.07 | 31.8 |
| SiWRKY015 | Sobic.002G202700 | 90.44 | 0.03 | 0.27 | 0.10 | 20.7 |
| SiWRKY017 | Sobic.002G355000 | 91.4 | 0.09 | 0.25 | 0.38 | 19.0 |
| SiWRKY018 | Sobic.002G418500 | 96.97 | 0.06 | 0.44 | 0.14 | 33.8 |
| SiWRKY019 | Sobic.003G248400 | 90.83 | 0.07 | 0.30 | 0.24 | 23.2 |
| SiWRKY020 | Sobic.009G034800 | 95.31 | 0.05 | 0.29 | 0.18 | 22.0 |
| SiWRKY022 | Sobic.003G037400 | 94.57 | 0.11 | 0.46 | 0.23 | 35.7 |
| SiWRKY023 | Sobic.004G138400 | 96 | 0.04 | 0.47 | 0.08 | 36.1 |
| SiWRKY024 | Sobic.009G247300 | 93.85 | 0.03 | 0.35 | 0.09 | 26.7 |
| SiWRKY025 | Sobic.010G045700 | 92.86 | 0.05 | 0.54 | 0.10 | 41.3 |
| SiWRKY027 | Sobic.003G248400 | 93.75 | 0.05 | 0.47 | 0.11 | 36.0 |
| SiWRKY028 | Sobic.009G212800 | 96.43 | 0.07 | 0.61 | 0.12 | 47.2 |
| SiWRKY029 | Sobic.009G206800 | 94.76 | 0.04 | 0.27 | 0.16 | 21.1 |
| SiWRKY032 | Sobic.001G083000 | 91.55 | 0.05 | 0.31 | 0.17 | 24.1 |
| SiWRKY033 | Sobic.009G100500 | 90.91 | 0.08 | 0.35 | 0.24 | 26.8 |
| SiWRKY039 | Sobic.002G174200 | 94.44 | 0.02 | 0.33 | 0.07 | 25.4 |
| SiWRKY042 | Sobic.004G065900 | 91.78 | 0.05 | 0.17 | 0.26 | 13.4 |
| SiWRKY043 | Sobic.003G138400 | 91.79 | 0.05 | 0.35 | 0.15 | 26.7 |
| SiWRKY044 | Sobic.003G040800 | 92.39 | 0.07 | 0.61 | 0.12 | 46.9 |
| SiWRKY046 | Sobic.003G037400 | 96 | 0.05 | 0.27 | 0.16 | 21.1 |
| SiWRKY049 | Sobic.010G045700 | 94.55 | 0.08 | 0.48 | 0.18 | 36.6 |
| SiWRKY051 | Sobic.003G199400 | 95 | 0.08 | 0.28 | 0.28 | 21.3 |
| SiWRKY052 | Sobic.003G248400 | 96.84 | 0.09 | 0.29 | 0.32 | 22.1 |
| SiWRKY054 | Sobic.003G226600 | 93.1 | 0.06 | 0.35 | 0.16 | 26.7 |
| SiWRKY062 | Sobic.003G341100 | 91.6 | 0.06 | 0.32 | 0.18 | 24.5 |
| SiWRKY064 | Sobic.003G444000 | 96.18 | 0.09 | 0.23 | 0.39 | 18.0 |
| SiWRKY070 | Sobic.007G085300 | 94.52 | 0.04 | 0.19 | 0.20 | 14.6 |
| SiWRKY076 | Sobic.009G087300 | 95.56 | 0.09 | 0.19 | 0.44 | 15.0 |
| SiWRKY080 | Sobic.005G014200 | 90.91 | 0.06 | 0.53 | 0.12 | 40.9 |
| SiWRKY082 | Sobic.008G029400 | 95.12 | 0.05 | 0.21 | 0.22 | 16.0 |
| SiWRKY095 | Sobic.001G055400 | 96.97 | 0.03 | 0.43 | 0.07 | 33.2 |
| SiWRKY096 | Sobic.009G171600 | 96.55 | 0.07 | 0.71 | 0.10 | 54.6 |
| SiWRKY097 | Sobic.001G084000 | 95.31 | 0.09 | 0.25 | 0.38 | 19.0 |
| SiWRKY098 | Sobic.008G153600 | 93.22 | 0.04 | 0.51 | 0.07 | 39.1 |
| SiWRKY099 | Sobic.001G148000 | 94.2 | 0.07 | 0.28 | 0.24 | 21.7 |
| SiWRKY100 | Sobic.001G148000 | 93.13 | 0.09 | 0.33 | 0.27 | 25.5 |
| SiWRKY103 | Sobic.003G048900 | 100 | 0.09 | 0.22 | 0.40 | 17.0 |
| SiWRKY105 | Sobic.001G389000 | 98.29 | 0.08 | 0.43 | 0.17 | 33.2 |
| **Mean** | | | **0.06** | **0.35** | **0.19** | **27.1** |
