# Supplementary material for: Global analysis of WRKY transcription factor superfamily in Setaria identifies potential candidates involved in abiotic stress signaling
Source: Front Plant Sci. 2015 Oct 26;6:910. doi: 10.3389/fpls.2015.00910 (PMC4654423; doi:10.3389/fpls.2015.00910)
Supplement: Supplementary file 8 [file Table8.DOC]

**Supplementary Table S8.** The Ka/Ks ratios and estimated divergence time for orthologous *WRKY* genes between *Setaria italica* and *Zea mays*.

| **Foxtail millet WRKY** | **Maize ortholog gene ID** | **% identity** | **Ka** | **Ks** | **Ka/Ks** | **Time of divergence (MYA)** |
| --- | --- | --- | --- | --- | --- | --- |
|
| SiWRKY001 | GRMZM2G040298_3 | 95.92 | 0.06 | 0.37 | 0.16 | 28.4 |
| SiWRKY002 | GRMZM2G169564_4 | 93.42 | 0.07 | 0.28 | 0.26 | 21.2 |
| SiWRKY003 | GRMZM2G036711_4 | 91.07 | 0.05 | 0.37 | 0.15 | 28.3 |
| SiWRKY004 | GRMZM2G040298_3 | 100 | 0.04 | 0.34 | 0.12 | 26.2 |
| SiWRKY005 | GRMZM2G377217_4 | 97.11 | 0.04 | 0.50 | 0.07 | 38.2 |
| SiWRKY006 | GRMZM2G138683_4 | 90.1 | 0.07 | 0.37 | 0.18 | 28.4 |
| SiWRKY008 | GRMZM5G812272_8 | 93.5 | 0.06 | 0.59 | 0.10 | 45.4 |
| SiWRKY010 | GRMZM2G377217_4 | 93.88 | 0.07 | 0.24 | 0.28 | 18.3 |
| SiWRKY014 | GRMZM2G125653_7 | 92.59 | 0.05 | 0.33 | 0.17 | 25.6 |
| SiWRKY015 | GRMZM2G125653_7 | 91.74 | 0.03 | 0.22 | 0.15 | 17.1 |
| SiWRKY016 | GRMZM2G052671_2 | 90.68 | 0.08 | 0.32 | 0.25 | 25.0 |
| SiWRKY019 | GRMZM5G812272_8 | 91.74 | 0.08 | 0.70 | 0.12 | 54.2 |
| SiWRKY020 | GRMZM5G851490_10 | 91.54 | 0.03 | 0.35 | 0.07 | 26.7 |
| SiWRKY022 | GRMZM2G475984_3 | 93.44 | 0.06 | 0.29 | 0.20 | 22.2 |
| SiWRKY023 | GRMZM2G073272_5 | 94.64 | 0.03 | 0.34 | 0.08 | 26.1 |
| SiWRKY024 | GRMZM2G054125_4 | 91.04 | 0.06 | 0.37 | 0.16 | 28.4 |
| SiWRKY025 | GRMZM2G063880_8 | 100 | 0.06 | 0.32 | 0.18 | 24.3 |
| SiWRKY027 | GRMZM2G453571_6 | 96.18 | 0.11 | 0.46 | 0.23 | 35.7 |
| SiWRKY028 | GRMZM2G137802_8 | 96.83 | 0.11 | 0.48 | 0.22 | 36.6 |
| SiWRKY029 | GRMZM2G401521_6 | 94.95 | 0.08 | 0.42 | 0.20 | 32.5 |
| SiWRKY030 | GRMZM2G163418_2 | 90.2 | 0.09 | 0.37 | 0.25 | 28.4 |
| SiWRKY031 | GRMZM2G034421_8 | 94.17 | 0.05 | 0.28 | 0.18 | 21.4 |
| SiWRKY032 | GRMZM2G169966_6 | 95.83 | 0.06 | 0.29 | 0.20 | 22.7 |
| SiWRKY033 | GRMZM2G012724_6 | 93.44 | 0.05 | 0.36 | 0.14 | 27.4 |
| SiWRKY035 | GRMZM2G314652_3 | 100 | 0.04 | 0.19 | 0.19 | 14.7 |
| SiWRKY038 | AC193630.3_FG003_9 | 94.37 | 0.05 | 0.51 | 0.10 | 39.3 |
| SiWRKY039 | GRMZM2G432583_8 | 97.5 | 0.03 | 0.35 | 0.09 | 27.1 |
| SiWRKY043 | GRMZM2G092694_8 | 93.39 | 0.11 | 0.26 | 0.43 | 20.3 |
| SiWRKY044 | GRMZM2G151444_3 | 94.65 | 0.06 | 0.37 | 0.16 | 28.4 |
| SiWRKY046 | GRMZM2G516301_8 | 96.9 | 0.07 | 0.28 | 0.26 | 21.2 |
| SiWRKY047 | GRMZM2G149683_8 | 96.3 | 0.05 | 0.37 | 0.15 | 28.3 |
| SiWRKY048 | AC198725.4_FG009_3 | 94.19 | 0.04 | 0.34 | 0.12 | 26.2 |
| SiWRKY049 | GRMZM2G411766_8 | 91.77 | 0.07 | 0.24 | 0.28 | 18.3 |
| SiWRKY052 | GRMZM2G453571_6 | 96.64 | 0.07 | 0.37 | 0.18 | 28.4 |
| SiWRKY053 | GRMZM2G137802_8 | 90.91 | 0.06 | 0.59 | 0.10 | 45.4 |
| SiWRKY054 | GRMZM2G149683_8 | 90.62 | 0.07 | 0.24 | 0.28 | 18.3 |
| SiWRKY055 | GRMZM2G111354_8 | 94.05 | 0.05 | 0.33 | 0.17 | 25.6 |
| SiWRKY056 | GRMZM2G013391_8 | 95.24 | 0.11 | 0.46 | 0.23 | 35.7 |
| SiWRKY057 | GRMZM2G030858_5 | 98.11 | 0.11 | 0.48 | 0.22 | 36.6 |
| SiWRKY060 | GRMZM2G158328_3 | 97.96 | 0.08 | 0.42 | 0.20 | 32.5 |
| SiWRKY061 | GRMZM2G059562_3 | 92.63 | 0.09 | 0.37 | 0.25 | 28.4 |
| SiWRKY062 | GRMZM5G822815_8 | 95.83 | 0.05 | 0.28 | 0.18 | 21.4 |
| SiWRKY064 | AC165171.2_FG002_3 | 90.29 | 0.06 | 0.29 | 0.20 | 22.7 |
| SiWRKY070 | GRMZM2G148561_4 | 94.52 | 0.06 | 0.26 | 0.22 | 19.9 |
| SiWRKY074 | GRMZM2G091331_10 | 94.16 | 0.07 | 0.24 | 0.28 | 18.3 |
| SiWRKY076 | GRMZM2G063216_4 | 91.53 | 0.03 | 0.34 | 0.08 | 26.1 |
| SiWRKY078 | GRMZM2G169564_4 | 93.42 | 0.05 | 0.42 | 0.11 | 32.5 |
| SiWRKY082 | GRMZM2G063216_4 | 93.75 | 0.11 | 0.25 | 0.43 | 19.5 |
| SiWRKY084 | GRMZM2G324999_1 | 97.22 | 0.06 | 0.37 | 0.16 | 28.4 |
| SiWRKY091 | GRMZM2G148087_3 | 97.83 | 0.12 | 0.28 | 0.44 | 21.3 |
| SiWRKY092 | GRMZM2G139815_7 | 91.04 | 0.07 | 0.30 | 0.24 | 22.8 |
| SiWRKY095 | GRMZM2G164082_1 | 96.38 | 0.08 | 0.32 | 0.25 | 24.2 |
| SiWRKY096 | GRMZM2G169966_6 | 94.83 | 0.08 | 0.47 | 0.18 | 36.1 |
| SiWRKY097 | GRMZM2G143204_1 | 94.77 | 0.07 | 0.24 | 0.28 | 18.3 |
| SiWRKY098 | GRMZM2G088096_9 | 97.14 | 0.07 | 0.37 | 0.18 | 28.4 |
| SiWRKY099 | GRMZM2G149219_1 | 95.24 | 0.06 | 0.59 | 0.10 | 45.4 |
| SiWRKY100 | GRMZM2G149219_1 | 92.59 | 0.07 | 0.51 | 0.15 | 38.9 |
| SiWRKY101 | GRMZM2G130374_1 | 100 | 0.05 | 0.33 | 0.17 | 25.6 |
| SiWRKY103 | GRMZM2G160554_1 | 98.43 | 0.03 | 0.22 | 0.15 | 17.1 |
| SiWRKY105 | GRMZM2G030272_1 | 96.03 | 0.07 | 0.24 | 0.28 | 18.3 |
| **Mean** | | | **0.07** | **0.36** | **0.19** | **27.5** |
