# Supplementary material for: Global analysis of WRKY transcription factor superfamily in Setaria identifies potential candidates involved in abiotic stress signaling
Source: Front Plant Sci. 2015 Oct 26;6:910. doi: 10.3389/fpls.2015.00910 (PMC4654423; doi:10.3389/fpls.2015.00910)
Supplement: Supplementary file 9 [file Table9.DOC]

**Supplementary Table S9.** The Ka/Ks ratios and estimated divergence time for orthologous WRKY proteins between *Setaria viridis* and *Panicum virgatum*.

| **Foxtail millet WRKY** | **Switchgrass ortholog gene ID** | **% identity** | **Ka** | **Ks** | **Ka/Ks** | **Time of divergence (MYA)** |
| --- | --- | --- | --- | --- | --- | --- |
|
| SvWRKY002 | Pavir.J40637.1.p | 97.47 | 0.03 | 0.09 | 0.33 | 6.8 |
| SvWRKY003 | Pavir.J40637.1.p | 80.95 | 0.02 | 0.04 | 0.40 | 3.3 |
| SvWRKY004 | Pavir.Gb01477.1.p | 92.11 | 0.02 | 0.05 | 0.35 | 3.8 |
| SvWRKY006 | Pavir.Ea02968.1.p | 85.93 | 0.03 | 0.05 | 0.54 | 4.0 |
| SvWRKY007 | Pavir.Ga01476.1.p | 84.62 | 0.02 | 0.05 | 0.38 | 3.5 |
| SvWRKY008 | Pavir.Ba00227.1.p | 85.71 | 0.02 | 0.06 | 0.26 | 5.0 |
| SvWRKY009 | Pavir.Ib02081.1.p | 89.26 | 0.13 | 0.06 | 2.21 | 4.4 |
| SvWRKY010 | Pavir.J08407.2.p | 81.15 | 0.13 | 0.06 | 2.05 | 4.7 |
| SvWRKY011 | Pavir.J09582.1.p | 83.33 | 0.02 | 0.07 | 0.24 | 5.0 |
| SvWRKY013 | Pavir.Eb01898.1.p | 89.73 | 0.05 | 0.07 | 0.81 | 5.2 |
| SvWRKY015 | Pavir.Aa00351.1.p | 86.84 | 0.05 | 0.07 | 0.81 | 5.2 |
| SvWRKY017 | Pavir.J39511.1.p | 81.47 | 0.05 | 0.07 | 0.77 | 5.4 |
| SvWRKY018 | Pavir.J08407.2.p | 81.82 | 0.05 | 0.07 | 0.75 | 5.6 |
| SvWRKY019 | Pavir.Ea00275.1.p | 84.47 | 0.02 | 0.07 | 0.28 | 5.7 |
| SvWRKY021 | Pavir.Ab02810.1.p | 84.76 | 0.13 | 0.07 | 1.84 | 5.4 |
| SvWRKY022 | Pavir.J08818.1.p | 88.71 | 0.04 | 0.07 | 0.63 | 5.0 |
| SvWRKY023 | Pavir.Eb03561.1.p | 80 | 0.09 | 0.06 | 1.53 | 4.7 |
| SvWRKY025 | Pavir.J12348.1.p | 95.77 | 0.06 | 0.06 | 0.99 | 4.4 |
| SvWRKY026 | Pavir.J01311.1.p | 84.92 | 0.03 | 0.05 | 0.50 | 4.1 |
| SvWRKY029 | Pavir.Gb00758.1.p | 88.48 | 0.01 | 0.05 | 0.21 | 4.1 |
| SvWRKY030 | Pavir.J03888.2.p | 100 | 0.03 | 0.05 | 0.50 | 4.1 |
| SvWRKY032 | Pavir.J12030.1.p | 85.33 | 0.06 | 0.05 | 1.19 | 3.7 |
| SvWRKY033 | Pavir.Ib03991.2.p | 98.28 | 0.02 | 0.05 | 0.49 | 3.7 |
| SvWRKY035 | Pavir.J08407.2.p | 83.82 | 0.05 | 0.05 | 0.99 | 4.1 |
| SvWRKY036 | Pavir.J40637.1.p | 96.83 | 0.01 | 0.06 | 0.15 | 4.7 |
| SvWRKY037 | Pavir.J14458.1.p | 83.33 | 0.02 | 0.07 | 0.37 | 5.0 |
| SvWRKY040 | Pavir.J30274.1.p | 92.74 | 0.02 | 0.05 | 0.44 | 4.2 |
| SvWRKY041 | Pavir.Eb03561.1.p | 100 | 0.09 | 0.06 | 1.44 | 5.0 |
| SvWRKY042 | Pavir.Gb01477.1.p | 82.91 | 0.03 | 0.05 | 0.50 | 4.1 |
| SvWRKY043 | Pavir.Gb01200.1.p | 88.79 | 0.09 | 0.07 | 1.29 | 5.2 |
| SvWRKY044 | Pavir.J04286.1.p | 85.17 | 0.09 | 0.07 | 1.21 | 5.5 |
| **Mean** | | | **0.05** | **0.06** | **0.79** | **4.7** |
