# Supplementary material for: Global analysis of WRKY transcription factor superfamily in Setaria identifies potential candidates involved in abiotic stress signaling
Source: Front Plant Sci. 2015 Oct 26;6:910. doi: 10.3389/fpls.2015.00910 (PMC4654423; doi:10.3389/fpls.2015.00910)
Supplement: Supplementary file 10 [file Table10.DOC]

**Supplementary Table S10.** The Ka/Ks ratios and estimated divergence time for orthologous WRKY proteins between *Setaria viridis* and *Sorghum bicolor*.

| **Foxtail millet WRKY** | **Sorghum ortholog gene ID** | **% identity** | **Ka** | **Ks** | **Ka/Ks** | **Time of divergence (MYA)** |
| --- | --- | --- | --- | --- | --- | --- |
|
| SvWRKY002 | Sobic.008G107500.1.p | 92.7 | 0.08 | 0.42 | 0.20 | 32.5 |
| SvWRKY004 | Sobic.002G355000.1.p | 88.24 | 0.09 | 0.37 | 0.25 | 28.4 |
| SvWRKY006 | Sobic.003G285500.1.p | 82.35 | 0.05 | 0.28 | 0.18 | 21.4 |
| SvWRKY007 | Sobic.002G202700.1.p | 81.82 | 0.06 | 0.29 | 0.20 | 22.7 |
| SvWRKY008 | Sobic.002G202800.2.p | 81.82 | 0.06 | 0.26 | 0.22 | 19.9 |
| SvWRKY009 | Sobic.001G332500.1.p | 85.2 | 0.07 | 0.24 | 0.28 | 18.3 |
| SvWRKY013 | Sobic.003G227300.1.p | 84.55 | 0.03 | 0.34 | 0.08 | 26.1 |
| SvWRKY015 | Sobic.010G035300.1.p | 81.54 | 0.05 | 0.42 | 0.11 | 32.5 |
| SvWRKY019 | Sobic.004G312200.1.p | 80.52 | 0.11 | 0.25 | 0.43 | 19.5 |
| SvWRKY026 | Sobic.003G000600.1.p | 80.52 | 0.06 | 0.37 | 0.16 | 28.4 |
| SvWRKY029 | Sobic.001G282400.1.p | 83.64 | 0.12 | 0.28 | 0.44 | 21.3 |
| SvWRKY030 | Sobic.006G201000.1.p | 84.62 | 0.07 | 0.30 | 0.24 | 22.8 |
| SvWRKY031 | Sobic.001G017100.1.p | 89.66 | 0.08 | 0.32 | 0.25 | 25.0 |
| SvWRKY032 | Sobic.003G248400.1.p | 80.49 | 0.08 | 0.70 | 0.12 | 54.2 |
| SvWRKY033 | Sobic.001G162100.1.p | 92.66 | 0.03 | 0.35 | 0.07 | 26.7 |
| SvWRKY035 | Sobic.001G084000.1.p | 80 | 0.06 | 0.29 | 0.20 | 22.2 |
| SvWRKY036 | Sobic.008G107500.1.p | 90.5 | 0.03 | 0.34 | 0.08 | 26.1 |
| SvWRKY040 | Sobic.006G206000.1.p | 89.06 | 0.06 | 0.37 | 0.16 | 28.4 |
| SvWRKY043 | Sobic.003G040800.1.p | 85.19 | 0.06 | 0.32 | 0.18 | 24.3 |
| SvWRKY044 | Sobic.002G242500.1.p | 80.72 | 0.11 | 0.46 | 0.23 | 35.7 |
| **Mean** | | | **0.07** | **0.35** | **0.21** | **26.8** |
