# Supplementary material for: Global analysis of WRKY transcription factor superfamily in Setaria identifies potential candidates involved in abiotic stress signaling
Source: Front Plant Sci. 2015 Oct 26;6:910. doi: 10.3389/fpls.2015.00910 (PMC4654423; doi:10.3389/fpls.2015.00910)
Supplement: Supplementary file 11 [file Table11.DOC]

**Supplementary Table S11.** The Ka/Ks ratios and estimated divergence time for orthologous WRKY proteins between *Setaria viridis* and *Zea mays*.

| **Foxtail millet WRKY** | **Maize ortholog gene ID** | **% identity** | **Ka** | **Ks** | **Ka/Ks** | **Time of divergence (MYA)** |
| --- | --- | --- | --- | --- | --- | --- |
|
| SvWRKY001 | GRMZM2G004060_P01 | 81.58 | 0.07 | 0.37 | 0.18 | 28.4 |
| SvWRKY002 | GRMZM2G143765_P01 | 92.06 | 0.03 | 0.22 | 0.15 | 17.1 |
| SvWRKY004 | GRMZM5G816457_P01 | 89.47 | 0.07 | 0.51 | 0.15 | 38.9 |
| SvWRKY005 | GRMZM2G064195_P01 | 94.74 | 0.05 | 0.33 | 0.17 | 25.6 |
| SvWRKY006 | GRMZM2G040298_P01 | 90.32 | 0.03 | 0.22 | 0.15 | 17.1 |
| SvWRKY007 | GRMZM2G125653_P02 | 81.82 | 0.08 | 0.32 | 0.25 | 25.0 |
| SvWRKY008 | GRMZM2G381378_P01 | 85.71 | 0.08 | 0.70 | 0.12 | 54.2 |
| SvWRKY009 | GRMZM2G171428_P02 | 82 | 0.03 | 0.35 | 0.07 | 26.7 |
| SvWRKY010 | GRMZM2G401521_P02 | 80.56 | 0.06 | 0.29 | 0.20 | 22.2 |
| SvWRKY011 | GRMZM2G354384_P01 | 90.91 | 0.03 | 0.34 | 0.08 | 26.1 |
| SvWRKY012 | GRMZM2G468056_P01 | 86.96 | 0.06 | 0.37 | 0.16 | 28.4 |
| SvWRKY013 | AC198725.4_FGP009 | 80.28 | 0.06 | 0.32 | 0.18 | 24.3 |
| SvWRKY025 | GRMZM2G106560_P02 | 94.44 | 0.11 | 0.46 | 0.23 | 35.7 |
| SvWRKY027 | GRMZM2G091331_P02 | 80 | 0.11 | 0.48 | 0.22 | 36.6 |
| SvWRKY030 | GRMZM2G013391_P01 | 86.44 | 0.08 | 0.42 | 0.20 | 32.5 |
| SvWRKY031 | GRMZM2G352415_P01 | 89.66 | 0.09 | 0.37 | 0.25 | 28.4 |
| SvWRKY032 | GRMZM2G151763_P01 | 84.42 | 0.11 | 0.26 | 0.43 | 20.3 |
| SvWRKY033 | GRMZM2G070211_P01 | 90.14 | 0.06 | 0.37 | 0.16 | 28.4 |
| SvWRKY036 | GRMZM2G076657_P02 | 90.5 | 0.07 | 0.28 | 0.26 | 21.2 |
| SvWRKY037 | GRMZM2G425430_P01 | 84.62 | 0.05 | 0.37 | 0.15 | 28.3 |
| SvWRKY038 | GRMZM2G354384_P01 | 90.91 | 0.04 | 0.34 | 0.12 | 26.2 |
| SvWRKY040 | GRMZM2G091331_P01 | 84.59 | 0.04 | 0.50 | 0.07 | 38.2 |
| SvWRKY043 | GRMZM2G123387_P02 | 96.12 | 0.03 | 0.22 | 0.15 | 17.1 |
| SvWRKY044 | GRMZM2G449681_P03 | 89.55 | 0.07 | 0.28 | 0.26 | 21.2 |
| **Mean** | | | **0.06** | **0.36** | **0.18** | **27.8** |
