# Supplementary material for: Global analysis of WRKY transcription factor superfamily in Setaria identifies potential candidates involved in abiotic stress signaling
Source: Front Plant Sci. 2015 Oct 26;6:910. doi: 10.3389/fpls.2015.00910 (PMC4654423; doi:10.3389/fpls.2015.00910)
Supplement: Supplementary file 12 [file Table12.DOC]

**Supplementary Table S12.** The Ka/Ks ratios and estimated divergence time for tandemly duplicated *SiWRKY* genes*.*

| **Gene 1** | **Chr** | **Start** | **End** | **Gene 2** | **Chr** | **Start** | **End** | **Distance (bp)** | **Ka** | **Ks** | **Ka/Ks** | **Time of divergence (MYA)** |
| --- | --- | --- | --- | --- | --- | --- | --- | --- | --- | --- | --- | --- |
| SiWRKY012 | 2 | 27207918 | 27209792 | SiWRKY013 | 2 | 27231842 | 27233604 | 22050 | 0.05 | 0.35 | 0.14 | 26.9 |
| SiWRKY032 | 3 | 16100904 | 16103618 | SiWRKY033 | 3 | 26202837 | 26205056 | 10099219 | 0.03 | 0.32 | 0.09 | 24.6 |
| SiWRKY040 | 4 | 23966320 | 23967446 | SiWRKY041 | 4 | 25418195 | 25419340 | 1450749 | 0.05 | 0.35 | 0.14 | 26.9 |
| SiWRKY058 | 5 | 40034269 | 40038719 | SiWRKY059 | 5 | 40040883 | 40043080 | 2164 | 0.06 | 0.34 | 0.18 | 26.2 |
| SiWRKY077 | 7 | 34186596 | 34187853 | SiWRKY078 | 7 | 34194819 | 34196066 | 6966 | 0.03 | 0.32 | 0.09 | 24.6 |
| SiWRKY078 | 7 | 34194819 | 34196066 | SiWRKY079 | 7 | 34202591 | 34204306 | 6525 | 0.04 | 0.33 | 0.12 | 25.4 |
| SiWRKY085 | 8 | 12904286 | 12905548 | SiWRKY086 | 8 | 12912885 | 12914076 | 7337 | 0.05 | 0.34 | 0.15 | 26.2 |
| SiWRKY086 | 8 | 12912885 | 12914076 | SiWRKY087 | 8 | 12959461 | 12960684 | 45385 | 0.05 | 0.35 | 0.14 | 26.9 |
| SiWRKY087 | 8 | 12959461 | 12960684 | SiWRKY088 | 8 | 12966027 | 12967302 | 5343 | 0.04 | 0.33 | 0.12 | 25.4 |
| SiWRKY099 | 9 | 9759533 | 9761173 | SiWRKY100 | 9 | 9767027 | 9768526 | 5854 | 0.05 | 0.34 | 0.15 | 26.2 |
| **Mean** | | | | | | | | | **0.05** | **0.34** | **0.13** | **25.9** |
