# Supplementary material for: Global analysis of WRKY transcription factor superfamily in Setaria identifies potential candidates involved in abiotic stress signaling
Source: Front Plant Sci. 2015 Oct 26;6:910. doi: 10.3389/fpls.2015.00910 (PMC4654423; doi:10.3389/fpls.2015.00910)
Supplement: Supplementary file 13 [file Table13.DOC]

**Supplementary Table S13.** The Ka/Ks ratios and estimated divergence time for segmentally duplicated *SiWRKY* genes*.*

| **Gene 1** | **Chr** | **Start** | **End** | **Gene 2** | **Chr** | **Start** | **End** | **Ka** | **Ks** | **Ka/Ks** | **Time of divergence (MYA)** |
| --- | --- | --- | --- | --- | --- | --- | --- | --- | --- | --- | --- |
| SiWRKY020 | 3 | 3470860 | 3472976 | SiWRKY043 | 5 | 3128175 | 3133163 | 0.03 | 0.32 | 0.09 | 24.6 |
| SiWRKY022 | 3 | 7209438 | 7210943 | SiWRKY046 | 5 | 11385866 | 11387183 | 0.29 | 0.04 | 0.14 | 22.3 |
| SiWRKY023 | 3 | 9059875 | 9061197 | SiWRKY047 | 5 | 29517728 | 29519669 | 0.03 | 0.3 | 0.1 | 23.1 |
| SiWRKY024 | 3 | 9086074 | 9088318 | SiWRKY048 | 5 | 29626945 | 29629320 | 0.02 | 0.31 | 0.06 | 23.9 |
| SiWRKY025 | 3 | 9758402 | 9760416 | SiWRKY049 | 5 | 31163423 | 31164962 | 0.04 | 0.29 | 0.14 | 22.3 |
| SiWRKY029 | 3 | 12588817 | 12589663 | SiWRKY055 | 5 | 36074399 | 36077406 | 0.04 | 0.31 | 0.13 | 23.9 |
| SiWRKY031 | 3 | 15637590 | 15640378 | SiWRKY057 | 5 | 40023634 | 40027402 | 0.29 | 0.03 | 0.1 | 22.3 |
| SiWRKY032 | 3 | 16100904 | 16103618 | SiWRKY062 | 5 | 40361245 | 40363952 | 0.02 | 0.27 | 0.07 | 20.8 |
| **Mean** | | | | | | | | **0.10** | **0.23** | **0.10** | **22.9** |
