# Supplementary material for: Global analysis of WRKY transcription factor superfamily in Setaria identifies potential candidates involved in abiotic stress signaling
Source: Front Plant Sci. 2015 Oct 26;6:910. doi: 10.3389/fpls.2015.00910 (PMC4654423; doi:10.3389/fpls.2015.00910)
Supplement: Supplementary file 14 [file Image1.PDF]

Supplementary Figure S1. Exon-Intron structure of SiWRKY genes.

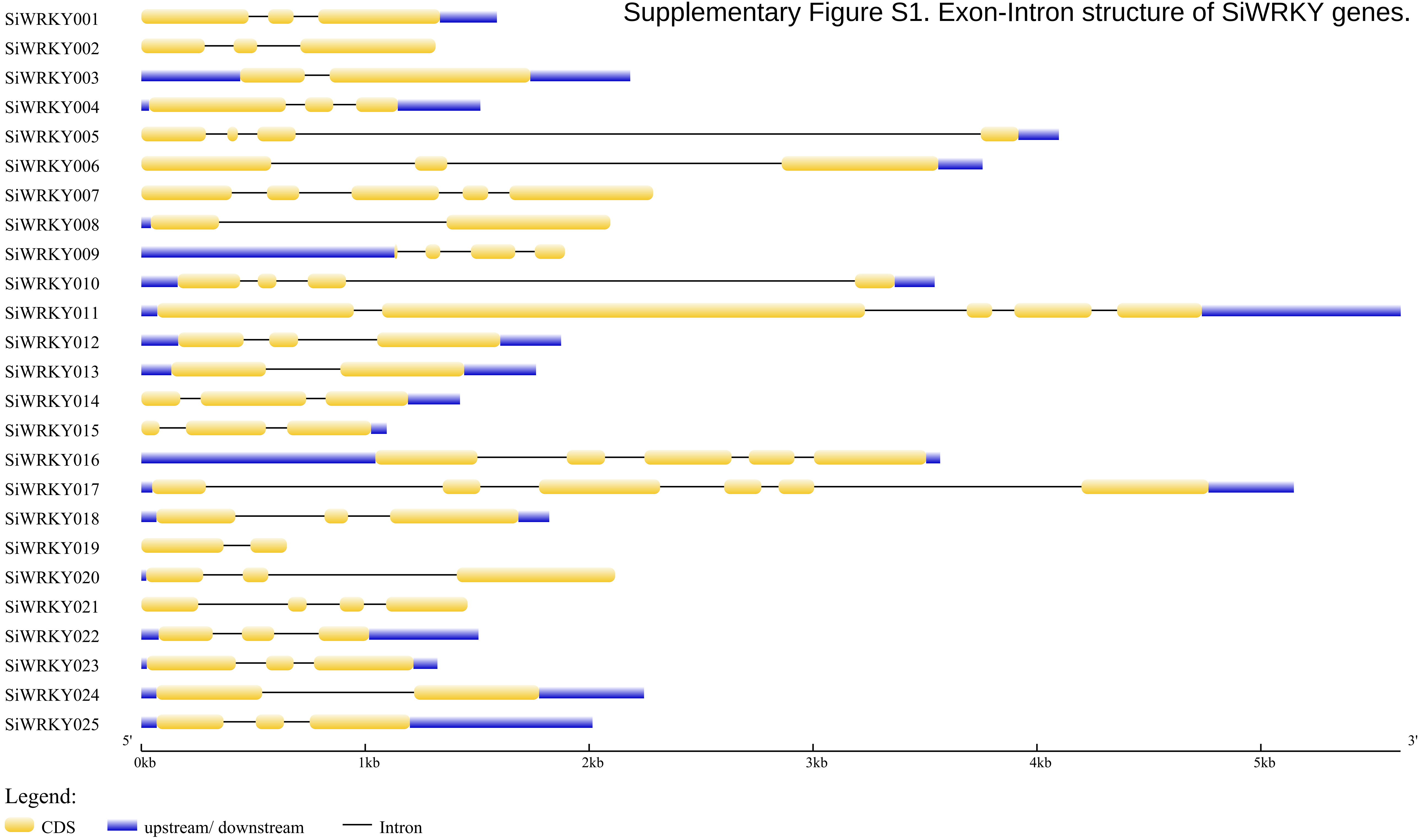

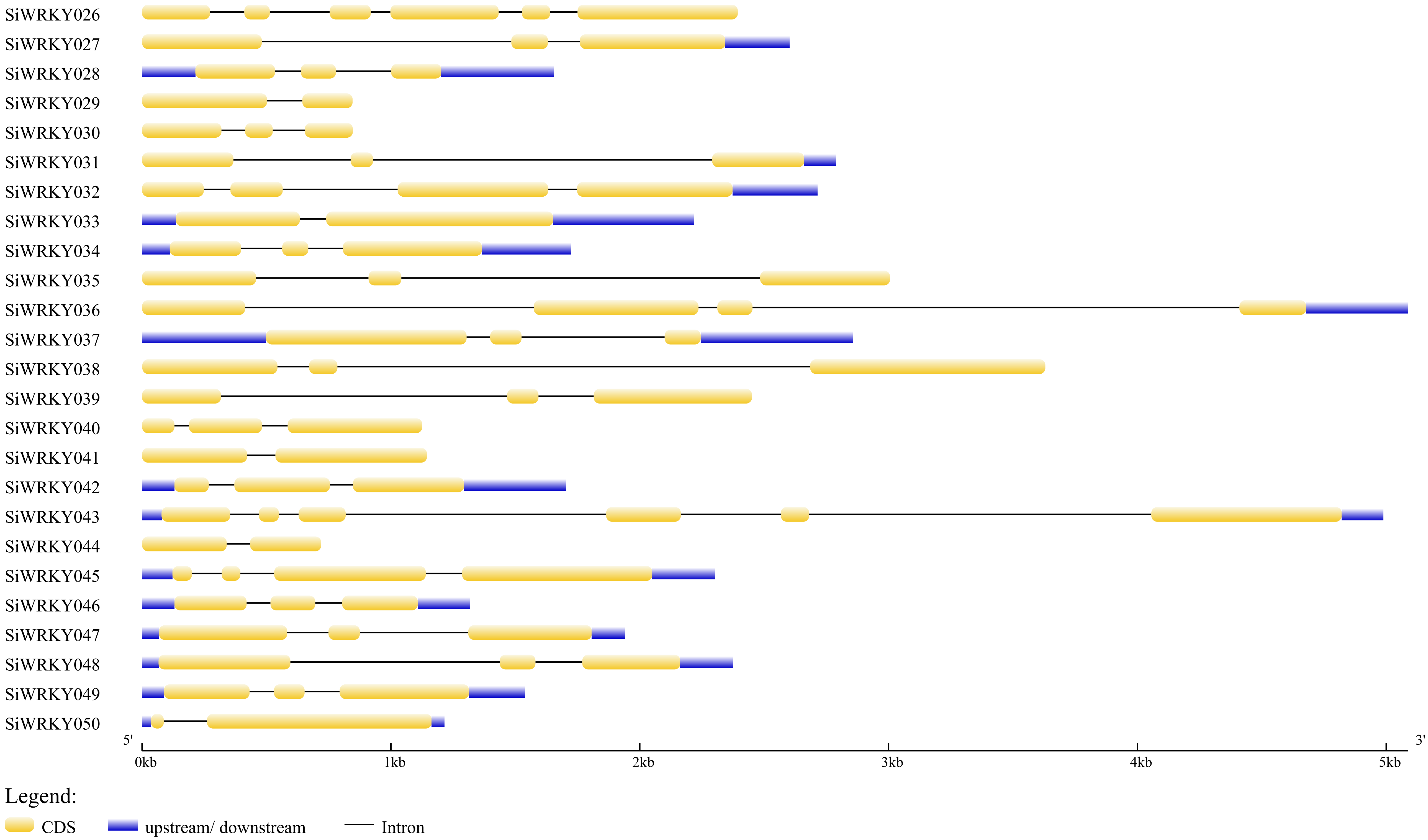
SiWRKY026
SiWRKY027
SiWRKY028
SiWRKY029
SiWRKY030
SiWRKY031
SiWRKY032
SiWRKY033
SiWRKY034
SiWRKY035
SiWRKY036
SiWRKY037
SiWRKY038
SiWRKY039
SiWRKY040
SiWRKY041
SiWRKY042
SiWRKY043
SiWRKY044
SiWRKY045
SiWRKY046
SiWRKY047
SiWRKY048
SiWRKY049
SiWRKY050
5'
3'
0kb
1kb
2kb
3kb
4kb
5kb
Legend:
CDS
upstream/ downstream
Intron

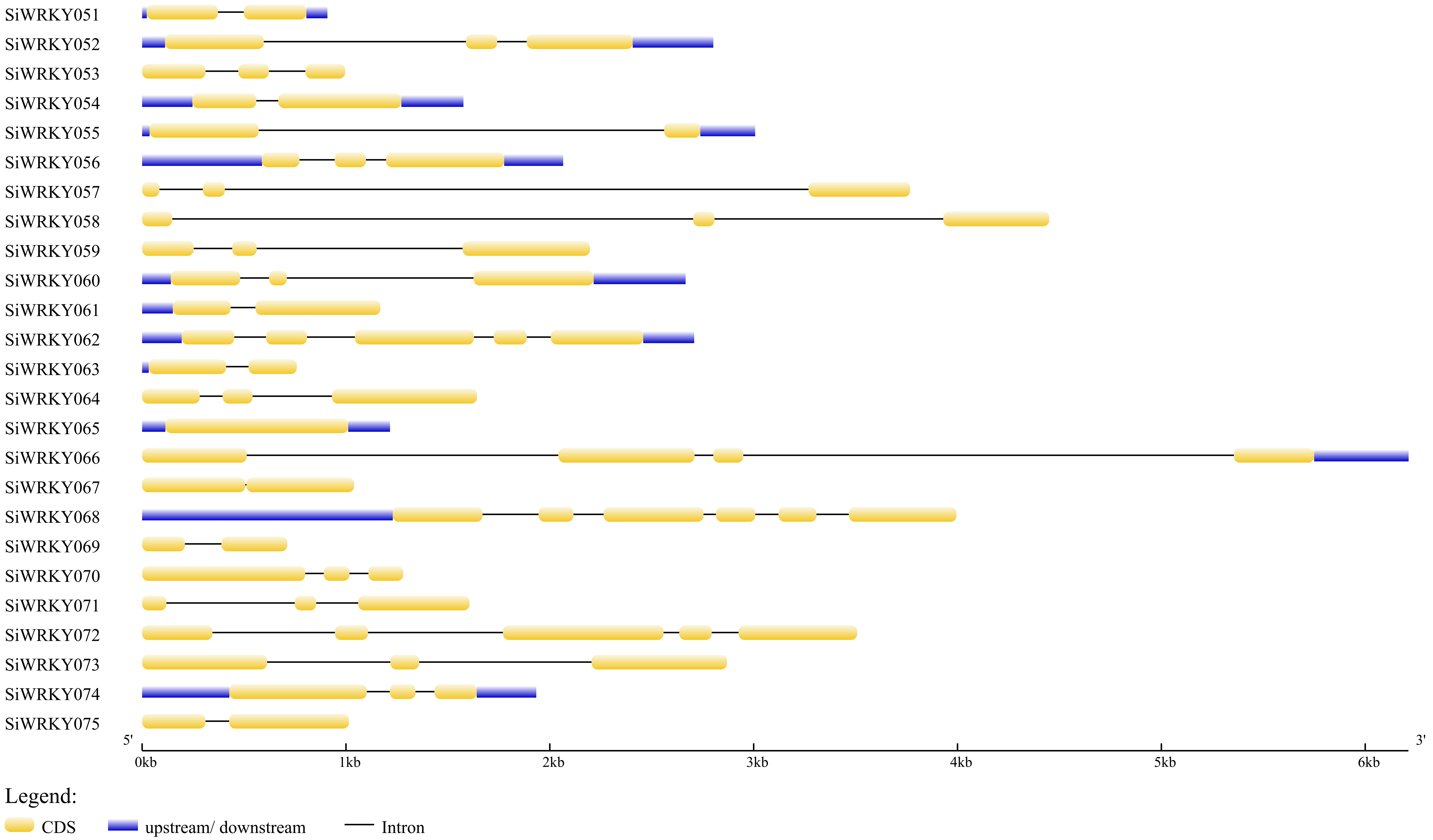
SiWRKY051
SiWRKY052
SiWRKY053
SiWRKY054
SiWRKY055
SiWRKY056
SiWRKY057
SiWRKY058
SiWRKY059
SiWRKY060
SiWRKY061
SiWRKY062
SiWRKY063
SiWRKY064
SiWRKY065
SiWRKY066
SiWRKY067
SiWRKY068
SiWRKY069
SiWRKY070
SiWRKY071
SiWRKY072
SiWRKY073
SiWRKY074
SiWRKY075
5'
3'
0kb
1kb
2kb
3kb
4kb
5kb
6kb
Legend:
CDS
upstream/ downstream
Intron

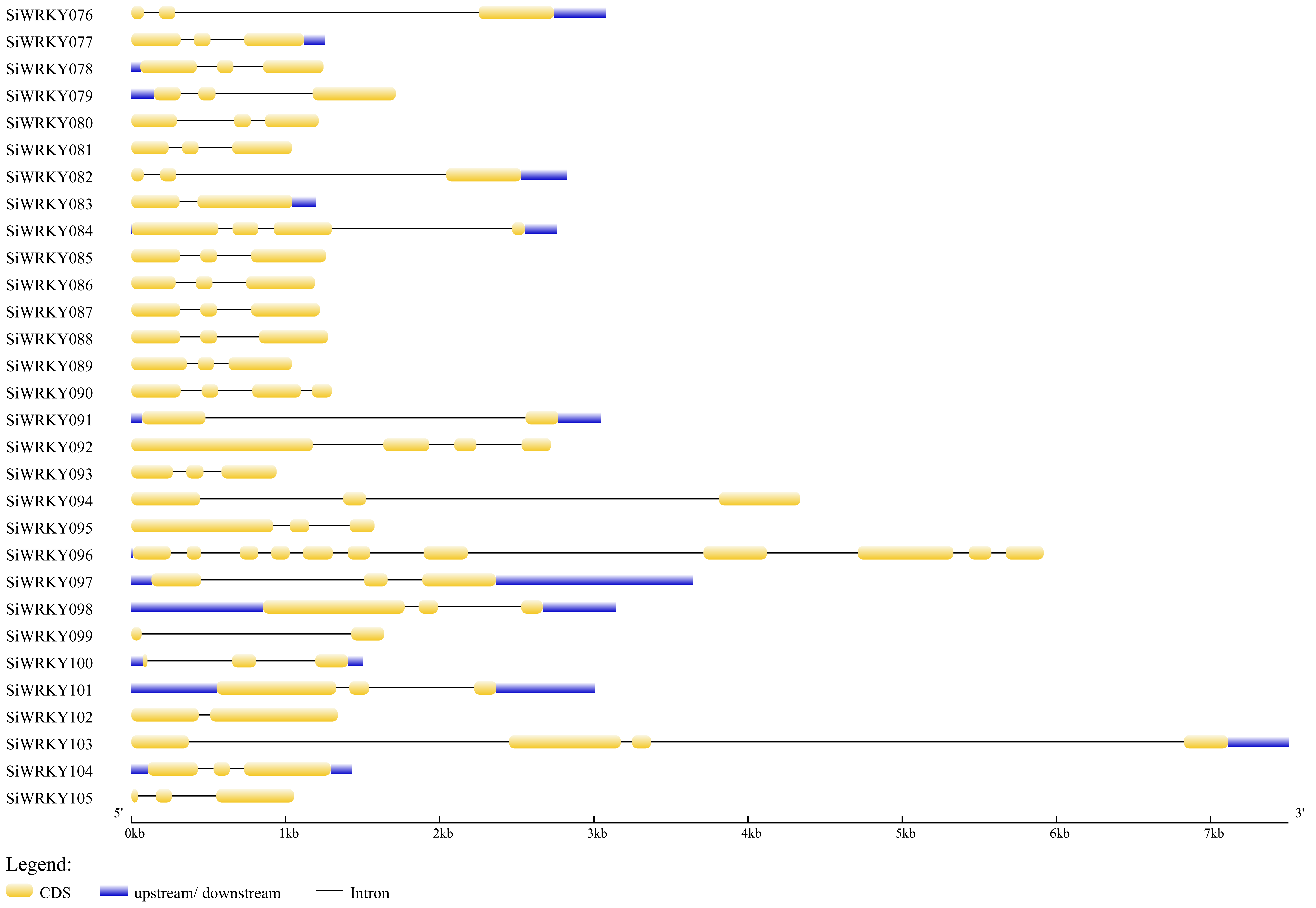
SiWRKY076
SiWRKY077
SiWRKY078
SiWRKY079
SiWRKY080
SiWRKY081
SiWRKY082
SiWRKY083
SiWRKY084
SiWRKY085
SiWRKY086
SiWRKY087
SiWRKY088
SiWRKY089
SiWRKY090
SiWRKY091
SiWRKY092
SiWRKY093
SiWRKY094
SiWRKY095
SiWRKY096
SiWRKY097
SiWRKY098
SiWRKY099
SiWRKY100
SiWRKY101
SiWRKY102
SiWRKY103
SiWRKY104
SiWRKY105
5'
3'
0kb
1kb
2kb
3kb
4kb
5kb
6kb
7kb
Legend:
CDS
upstream/ downstream
Intron
