# Supplementary material for: Global analysis of WRKY transcription factor superfamily in Setaria identifies potential candidates involved in abiotic stress signaling
Source: Front Plant Sci. 2015 Oct 26;6:910. doi: 10.3389/fpls.2015.00910 (PMC4654423; doi:10.3389/fpls.2015.00910)

Supplementary Figure S2. Relative expression values of *SiWRKY* genes in *Setaria italica* seedlings exposed to dehydration, salinity, abscisic acid (ABA), salicylic acid (SA) and methyl jasmonate (MeJA) treatments presented as bar diagram. *Act2* was used as an internal control to normalize the data. The error bars representing standard deviation were calculated based on three technical replicates for biological duplicate.

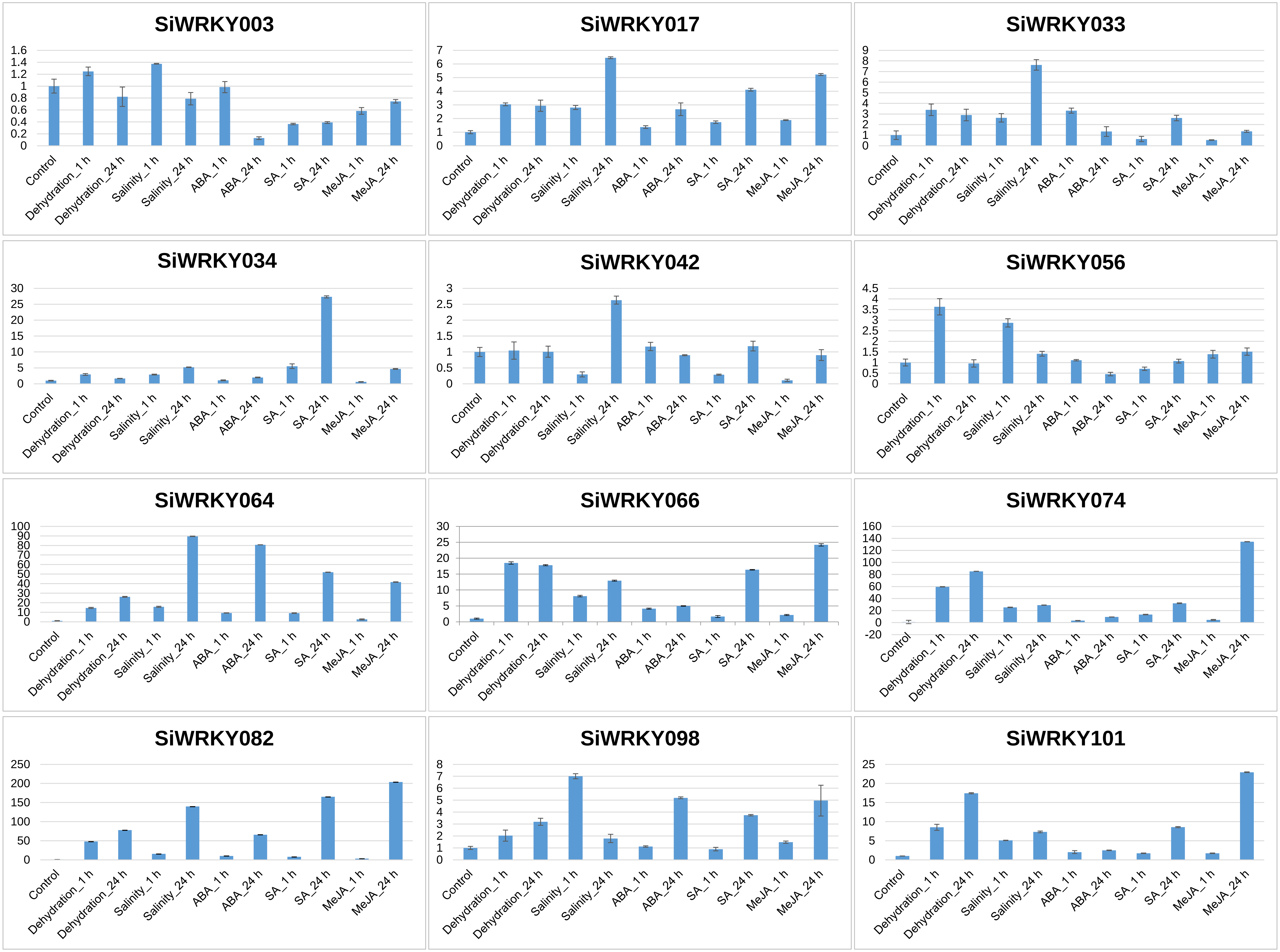

Supplement: Supplementary file 15 [file Image2.PDF]
