# Supplementary figures and images for: Global analysis of WRKY transcription factor superfamily in Setaria identifies potential candidates involved in abiotic stress signaling
Source: Front Plant Sci. 2015 Oct 26;6:910. doi: 10.3389/fpls.2015.00910 (PMC4654423; doi:10.3389/fpls.2015.00910)

Supplementary Figure S3. WRKY genes present in sequenced plant genomes

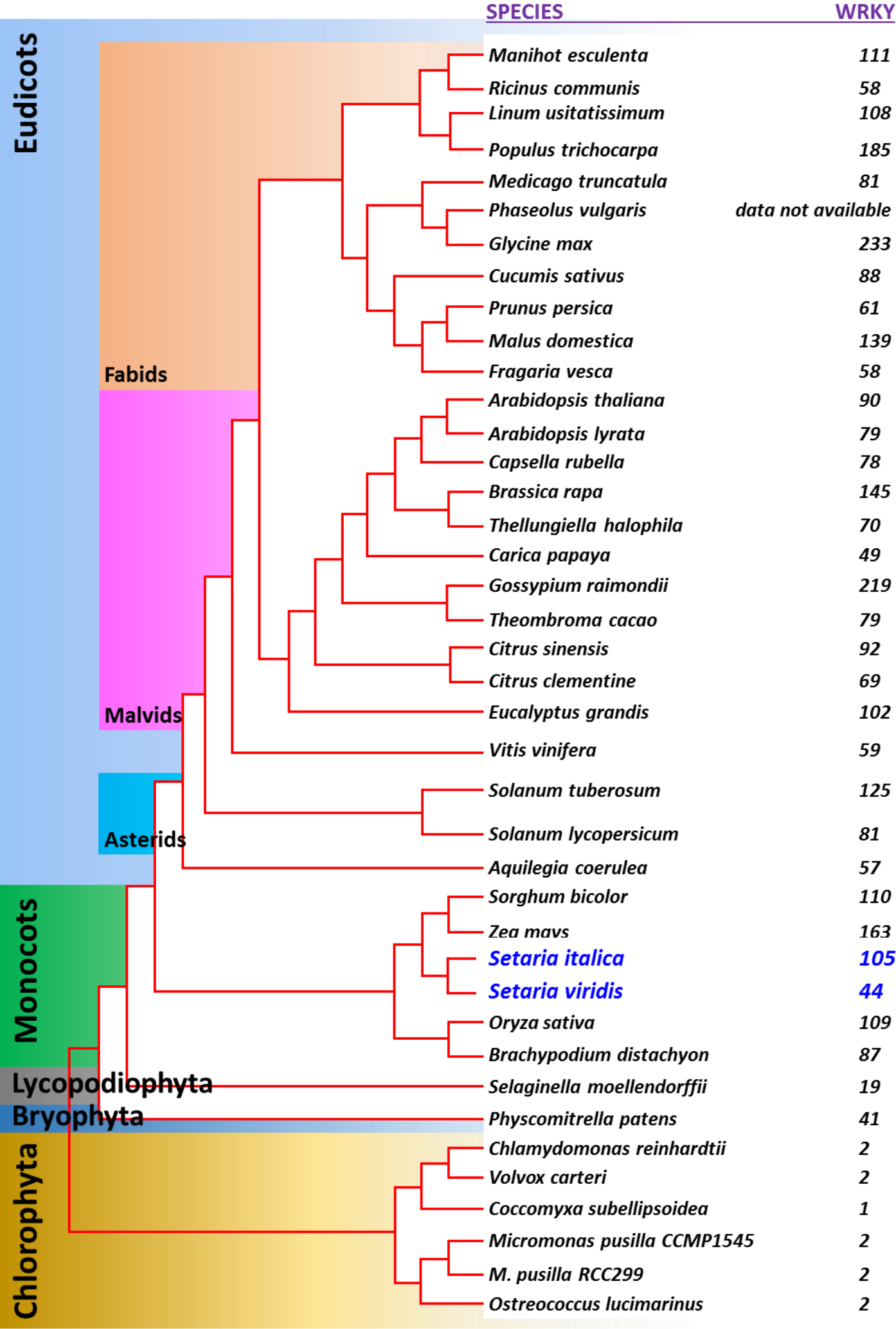

Supplement: Supplementary file 16 [file Image3.PDF]
